# Patterns in bird and pollinator occupancy and richness in a mosaic of urban office parks across scales and seasons

**DOI:** 10.1002/ece3.10958

**Published:** 2024-03-01

**Authors:** Kelly J. Iknayan, Sacha K. Heath, Scott B. Terrill, Daniel G. Wenny, Stephanie Panlasigui, Yiwei Wang, Erin E. Beller, Erica N. Spotswood

**Affiliations:** ^1^ San Francisco Estuary Institute Richmond California USA; ^2^ H. T. Harvey & Associates Los Gatos California USA; ^3^ San Francisco Bay Bird Observatory Milpitas California USA; ^4^ Real Estate and Workplace Services Sustainability Team, Google Inc. Mountain View California USA; ^5^ Second Nature Oakland California USA

**Keywords:** cities, landscape, local, multi‐species occupancy, neighborhood, species richness

## Abstract

Urbanization is a leading cause of global biodiversity loss, yet cities can provide resources required by many species throughout the year. In recognition of this, cities around the world are adopting strategies to increase biodiversity. These efforts would benefit from a robust understanding of how natural and enhanced features in urbanized areas influence various taxa. We explored seasonal and spatial patterns in occupancy and taxonomic richness of birds and pollinators among office parks in Santa Clara County, California, USA, where natural features and commercial landscaping have generated variation in conditions across scales. We surveyed birds and insect pollinators, estimated multi‐species occupancy and species richness, and found that spatial scale (local, neighborhood, and landscape scale), season, and urban sensitivity were all important for understanding how communities occupied sites. Features at the landscape (distance to streams or baylands) and local scale (tree canopy, shrub, or impervious cover) were the strongest predictors of avian occupancy in all seasons. Pollinator richness was influenced by local tree canopy and impervious cover in spring, and distance to baylands in early and late summer. We then predicted the relative contributions of different spatial scales to annual bird species richness by simulating “good” and “poor” quality sites based on influential covariates returned by the previous models. Shifting from poor to good quality conditions locally increased annual avian richness by up to 6.8 species with no predicted effect on the quality of the neighborhood. Conversely, sites of poor local and neighborhood scale quality in good‐quality landscapes were predicted to harbor 11.5 more species than sites of good local‐ and neighborhood‐scale quality in poor‐quality landscapes. Finally, more urban‐sensitive bird species were gained at good quality sites relative to urban tolerant species, suggesting that urban natural features at the local and landscape scales disproportionately benefited them.

## Graphical abstract

We explored seasonal and spatial patterns in bird and pollinator communities among urban office parks in Santa Clara County, California, USA, where natural features, and commercial landscaping aimed at biodiversity enhancement, have generated variation in conditions across scales. We surveyed birds and insect pollinators, estimated multi‐species occupancy and species richness, and found that spatial scale, season, and urban sensitivity were all important for understanding how communities occupied sites. Our results imply that both top‐down approaches that plan for biodiversity enhancements at the landscape‐scale as well as bottom‐up approaches that improve conditions at the local scale can work together to support birds and pollinators in cities.

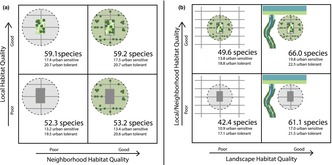

## 1 INTRODUCTION

Although urbanization has contributed to the global loss of natural heritage (McDonald et al., 2009; Shochat et al., 2010), surprisingly high levels of biodiversity have been reported in some cities (Faeth et al., 2011; Kowarik, 2011; McKinney, 2008; Spotswood et al., 2021). There are compelling reasons to restore nature to cities given the myriad benefits that an ecologically functioning city can provide including cleaner air and water, temperature modulation, increases in residents' well‐being, and improved outdoor experiences (Mata et al., 2020). There is also a growing interest in the role cities can play in conserving biodiversity, and cities around the world are adopting plans, developing strategies and targets, and developing approaches and indicators to measure outcomes (Pierce et al., 2020). These efforts would benefit from a robust understanding of the effect that habitat enhancement in cities can have on the occupancy and species richness of target taxonomic groups. Yet, many taxa respond to conditions at multiple spatial scales as well as seasonally, and this complexity has made it difficult to predict how urban greening actions may affect different species. Results from research that tackles this complexity can be used to inform both city‐scale planning efforts as well as small‐scale site interventions on public and private land.

Many taxonomic groups of urban wildlife, including birds and pollinators, are highly mobile, and factors at multiple spatial scales can influence their species richness (Aronson et al., 2017; Norton et al., 2016). At the landscape scale, distance to regionally important resources such as large water bodies (Barbosa et al., 2020), stream corridors (Beaugeard et al., 2021), and urban boundaries or large patches of habitat (Villaseñor et al., 2021) can be important either despite or in addition to local conditions. Neighborhood scale conditions are also important, with tree canopy cover (Lerman et al., 2021), the number of retained mature street trees (Barth et al., 2015), and native plant composition (Lerman & Warren, 2011) influencing bird species richness or abundance. Poor habitat conditions in a surrounding neighborhood (e.g., high proportions of impervious surface; Lerman et al., 2021) can have a negative impact on species richness even when local site conditions are beneficial (Ikin et al., 2013; Villaseñor et al., 2021). Local site features demonstrated to have positive associations with avian richness include habitat heterogeneity (Lerman et al., 2021), patch size (Callaghan et al., 2018; Daniels & Kirkpatrick, 2006), canopy height and shrub cover (Daniels & Kirkpatrick, 2006), tree cover (Ferenc et al., 2014), and understory volume (Threlfall et al., 2017). Pollinators appear most sensitive to floral resources and herbaceous plant species richness (Wilson & Jamieson, 2019). Finally, sensitivity to the urban landscape might drive how species respond to urban greening interventions, but results are mixed, with species groups with different urban tolerances responding differently to small‐scale habitat improvements in some cases (e.g., Lerman et al., 2021) but not in others (e.g., Archibald et al., 2017). Quantifying the relative magnitude of the effects of site, neighborhood, and regional scale conditions on urban birds and pollinators has the potential to shed light on both the opportunities and limitations of greening actions.

Birds and insect pollinators also have resource requirements and population dynamics that can vary throughout the year (e.g., Chen et al., 2018, 2019), suggesting that the effect strengths of urban habitat interventions at different scales may also vary seasonally. For example, urban green spaces are used seasonally as stopover sites by some migratory species (Pennington et al., 2008; Seewagen et al., 2010; Seewagen & Slayton, 2008), and migrants in transit may be more likely to respond to landscape scale conditions such as proximity to regional water bodies or large parks (Seewagen et al., 2010). On the other hand, breeding birds may respond most strongly to local conditions, particularly small‐sized territorial landbirds that depend on insects or other resources within small home ranges and who defend those spaces against other individuals (Chen et al., 2018; Schoener, 1968). We might also expect that year‐round residents who vary their resource requirements and home range sizes throughout the year (e.g., diet switching from insects to grains and fruits) may respond to a combination of site and neighborhood factors, depending on the season (Chen et al., 2019). Seasonal studies in urban areas to date have mostly focused on comparisons across the rural–urban gradient, for example, comparing whether urban communities are less temporally dynamic than their rural counterparts (La Sorte et al., 2014; Leveau & Leveau, 2016). Relatively few studies assess whether the habitat needs of urban wildlife vary seasonally (but see La Sorte et al., 2020). Understanding how bird occupancy and pollinator richness responds to environmental conditions throughout the annual cycle can inform planning and design at multiple scales, enabling a city to be ecologically diverse year‐round.

Here we use a comprehensive and unique dataset to seasonally explore the scale of habitat interventions and extant natural features that matter most for bird and pollinator communities in the low‐density corporate office parks of Silicon Valley, California, USA, where commercial landscaping aimed at biodiversity enhancement around buildings and in parking lots creates variation in site and neighborhood conditions. These properties represent an intermediate management scale between larger greenspaces and smaller privately held elements in the urban matrix, such as residential yards. Though privately held non‐residential land use typically occupies 20%–40% of a city's area (Rodrigue, 2020), this type of land use is rarely targeted for biodiversity enhancement or follow‐up studies to evaluate effectiveness. As the baseline for a planned longer‐term monitoring effort, we surveyed birds and insect pollinators and estimated occupancy and species richness across a spectrum of more natural to highly developed commercial sites throughout the year. We considered three key scales of influence and investigated how patterns in occupancy and richness were associated with (1) variation in the amount, complexity, and nativeness of local‐scale vegetation, (2) the amount of natural habitat cover in the neighborhood, and (3) the proximity to key natural features in the landscape. We also asked whether there was alignment in how birds and insect pollinator species occurred on the landscape.

For birds, we examined (1) the relative importance of landscape, neighborhood, and local scale environmental characteristics to species occupancy and richness, (2) whether the importance of environmental characteristics varied seasonally, and (3) whether sites with high species richness were composed of urban‐sensitive or urban‐adapted species. For insect pollinators, we asked whether pollinator taxonomic richness was associated with the same environmental variables as birds and whether this response varied seasonally. We expected that local‐scale factors would have greater effect sizes than landscape and neighborhood scale factors and that birds and pollinators would respond positively to local‐scale vegetative cover, complexity, and nativity, as per findings of other urban ecology studies (Chace & Walsh, 2006; Evans et al., 2009). We also expected that site characteristics associated with high levels of richness and occupancy would do so throughout the annual cycle. Using these findings, we provide local, neighborhood, and landscape scale management and planning recommendations to guide the creation of urban landscapes more likely to enhance the occupancy and richness of birds and insect pollinators throughout the year.

## 2 MATERIALS AND METHODS

### 2.1 Study area, sites, and species

**FIGURE 1:**
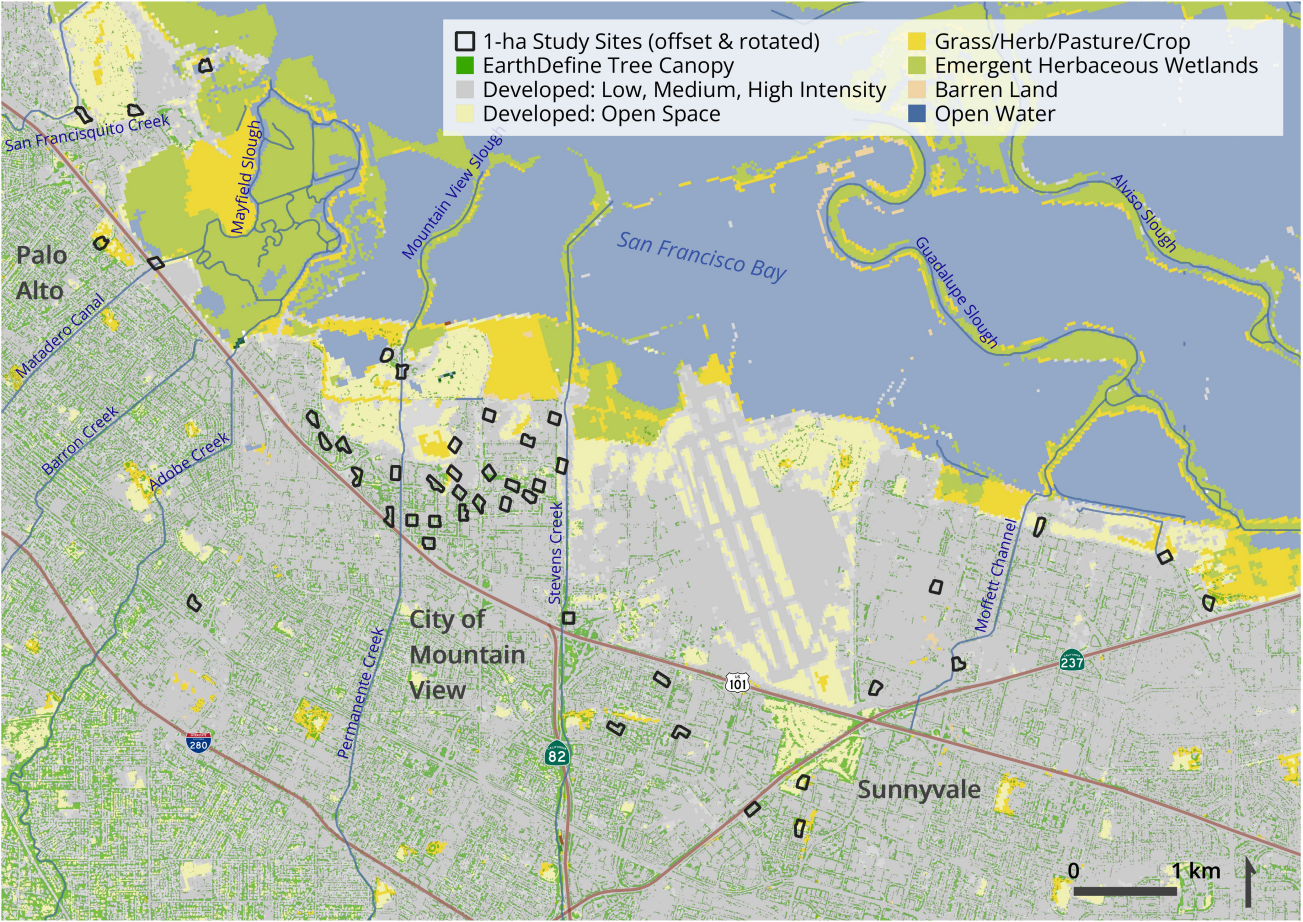
Study area and study sites (*n* = 45, outlined black) for avian and pollinator surveys in Santa Clara County of the south San Francisco Bay Area, California, USA. Random spatial jitter and rotation were used on study sites to protect the privacy of private landholders. Map created with the 30‐m‐resolution 2016 National Land Cover Database (Homer et al., 2020) and the 60‐cm‐resolution Tree Canopy layer (EarthDefine, 2013).

Surveys of avian and pollinator communities were conducted at 45 1‐ha sites located in three cities—Palo Alto, The City of Mountain View, and Sunnyvale—in the Santa Clara Valley of the south San Francisco Bay Area, California (Figure 1). This area is known colloquially as ‘Silicon Valley,’ famous for high‐tech companies and start‐ups. Santa Clara Valley experienced rapid urbanization in the late 20th century, with commercial and office park developments concentrated near San Francisco Bay in areas developed later than adjacent uplands due to higher groundwater levels (Beller et al., 2020; Grossinger et al., 2007). The resulting study area is a gradient mosaic of land use bounded in the north by tidal marsh mudflats and salt ponds of the Bay; moving southward through a band of semi‐natural vegetation of varying quality and comprising private and protected open space, a golf course, airfield, and decommissioned landfills; and ending in the south among office parks and medium‐ to high‐density human residential and small business areas with varying degrees of vegetative cover (Figure 1). Four primary creeks (San Francisquito, Adobe, Permanente, and Stevens creeks) and a few canals and sloughs run through the study area, all of which drain to the bay. Varying amounts of natural vegetation occur in and adjacent to the channels or on top of the bordering levees. The study area is characterized by a Mediterranean climate, with the region receiving 250 to 500 mm of precipitation annually, with warm, dry summers and cooler, wet winters during which the majority of rainfall occurs (McKee et al., 2003).

Most (82%) of the 1‐ha study sites were characterized entirely by proportions of the four land cover types (Open Space Developed and/or Low, Medium, or High‐Intensity Developed) developed by Homer et al. (2020). The remaining eight sites additionally included proportions of other land cover types (Cultivated Crops, Grassland/Herbaceous, Emergent Herbaceous Wetland, and/or Open Water). Sites varied by the proportions of impervious surface cover (e.g., parking lots, portions of corporate campuses) and different types of vegetated landscaping. These urban sites were embedded in neighborhoods that ranged from corporate campuses to more natural areas (e.g., city parks, urban nature preserves, golf courses, and riparian areas), and varied in their distances to the San Francisco Bay and urban streams in the wider landscape. Sites were on average 3.48 km apart (standard deviation ±2.48 km, range 0.14 to 11.82 km), and were irregularly shaped because they were defined by parcel boundaries and excluded building footprints. The 1‐ha sites were selected for sampling because of the anticipated addition of native plantings in future years; thus sites during the years of this study (2015–2017) also represented the baseline conditions for a long‐term monitoring study. Birds and pollinators were selected for study because native landscaping was and will be targeted with the requirements of birds and pollinators in mind; the expectation is that surveys of these taxa will continue to monitor bird and pollinator responses to the native plant enhancements over time.

### 2.2 Bird surveys

Avian field surveys were performed from winter 2015 through fall 2016 during four seasons relevant to the occupancy of breeding and non‐breeding birds in the study area: overwinter (November 2015 to January 2016), spring migration (March to May 2016), summer breeding (May to July 2016), and fall migration (August to October 2016). Sites were surveyed 8 to 9 times per season, at approximately 1‐week intervals, using 20‐min passive area searches during which all birds within the survey site were recorded (Loyn, 1986; Slater, 1994). This time‐ and area‐constrained method is recommended for habitat patches of small size that will not allow for the placement of multiple independent point counts. Area searches allow for movement around a site and were originally devised for winter surveys when birds are mostly detected visually (vs. during the breeding season when birds are mostly detected aurally; Twedt et al., 2008). Estimates derived from this method are comparable and sometimes higher than line transects or strip transects (Roberts & Schnell, 2006; Watson, 2004). Surveys were completed within 4 h of sunrise. Due to construction activities, not all 45 sites were surveyed each season, resulting in 35 to 44 sites surveyed per season.

### 2.3 Pollinator surveys

Visual surveys of insect pollinators were conducted from spring to late summer of 2017. Seasons were selected to capture the seasonal variation in floral resources and pollinator activity: spring (April 27 to May 2), early summer (May 31 to June 2), and late summer (August 31). We placed one pollinator survey plot of 100 m^2^ within each of the larger 1‐ha sites and sampled the same plot during each visit. We selected the plot location in vegetated areas with floral resources that we assumed would be most attractive to pollinators. Thus the plots were not representative of the 1‐ha plots which were often characterized by largely unvegetated areas such as parking lots and paved meeting areas, and instead were representative of vegetation patches with the highest floral resources within the 1‐ha sites. Due to construction activities, not all sites were accessible during every season, and surveys were conducted 1 to 3 times per site (mean: 2.57 visits). Fifteen‐minute visual surveys occurred between 10:00 and 15:00. A practiced entomologist—with experience in visually identifying most regional floral insect visitors by sight—conducted all pollinator surveys. The surveyor followed a meandering transect through the plot and recorded all encountered invertebrates located on the reproductive parts of flowers. In the interest of not depleting native pollinators, the surveyor visually identified invertebrates to the lowest feasible taxonomic level. Dipterans were identified to family, Hymenopterans were identified to genera, and Lepidopterans to species except for *Hesperia* sp., *Hylephila* sp., and *Phyciodes* sp. To confirm visual identification of the most commonly encountered cryptic species, a sample individual was hand‐captured and identified in the lab using a dissecting scope, an independently confirmed reference collection, and a definitive reference book by Michener (2000). For the complete list of taxa (*n* = 42), see Table S1 in Appendix S1.

### 2.4 Environmental variables

Environmental variables were calculated at three spatial scales: local, neighborhood, and landscape (Table S2 in Appendix S1). Local‐scale variables were derived from data collected at 1‐ha sites during the summer of 2016 to characterize vegetation composition, land cover, and vertical vegetation complexity. These data represent direct measures of vegetation cover and complexity in the summer. Because we did not collect vegetation data in other seasons, however, we made the assumption that differences between sites during fall, winter, and spring are represented by differences between sites during the summer. In 1‐ha plots, we counted and identified (to at least genus level) all native and non‐native trees (where trees are defined as woody plants with trunks ≥10 cm diameter at breast height). From these counts, we calculated the ratio of native to non‐native trees. Within each 1‐ha site, we used GIS to randomly select six 10 × 5 m subplots. Within each subplot, we visually estimated the percentage of ground cover for impervious surface and each native and non‐native herbaceous, tree, and shrub species independently in 1% increments and 0.1% placeholders for trace quantities of a given cover type. Vertical vegetation complexity was assessed using a 1 × 6‐foot cover board, divided into six one‐foot vertical bands, following Herrick et al. (2009, pp. 61–62). Measurements were taken 4.5 m away from the observer in each cardinal direction. Bands were considered obscured if >25% were covered in vegetation. The number of obscured bands was divided by the total number of bands and averaged across all measures taken at the site. Values from the six subplots were averaged and considered representative of the 1‐ha site as a whole.

We quantified neighborhood‐scale variables (tree canopy and impervious cover) within 500 m buffers around sites using ArcGIS (ESRI, 2020). We chose this buffer distance because it is the minimum value of the range of most commonly used distance buffers (500–2500 m) from which variables significantly associated with birds have been derived (Litteral & Shochat, 2017). It is also a distance buffer shown to be relevant to urban insects (Turrini & Knop, 2015). The canopy cover was derived from Earth Define (2013) 60‐cm‐resolution Tree Canopy data set, and the impervious cover was derived from the 30‐m‐resolution 2016 National Land Cover Database (Homer et al., 2020).

We calculated landscape‐scale variables to characterize the site's position relative to water features. Distance to the San Francisco Baylands (i.e., the closest tidal bay flats or open bay from the site edge) was measured using the California Aquatic Resource Inventory (SFEI, 2017a). Distance to the nearest stream or channel was calculated using the Bay Area Aquatic Resources Inventory (SFEI, 2017b).

### 2.5 Occupancy models

We used robust‐design, dynamic, multi‐species occupancy models to evaluate species‐level occupancy and community composition of birds across a range of urban conditions while correcting for imperfect detection (Iknayan et al., 2014). We temporally partitioned the sampling occasions in each season into three primary sampling periods each comprising triplet adjacent samples. The model assumes closure within the secondary sampling periods but permits changes in occupancy between primary periods (Altwegg & Nichols, 2019; MacKenzie et al., 2003; Pollock, 1982). We modeled each season independently with single‐season occupancy models rather than linked multi‐season models (Dorazio et al., 2010). Since many species enter or leave the study area between seasons, a multiseason model would capture migratory behaviors for many species rather than extinction and colonization dynamics driven by site suitability. Further, the multiseason approach requires the same species list to be used across all seasons, including those not available for sampling, which could introduce additional uncertainty in parameter estimation. Only species that were present in at least one site during that season were included in that season's model.

In our study, we define the term “occupancy” as the use of a site at any point during the sampling season. A core assumption of occupancy models is that sites experience no species immigration or emigration during a season (i.e., the “closure assumption”). Violating the closure assumption can overestimate occupancy when detection probabilities are low, potentially leading to inflated species richness estimates (MacKenzie et al., 2018, 146). Given the dynamic migratory behavior of birds in spring and fall, specific subsets of species, particularly those with transient behavior, could wield relatively greater influence on covariate estimations through inflation of their estimated occupancy. To further accommodate the migration of species during the course of a season beyond the use of a dynamic model with secondary sampling periods, we allowed variation of detection probabilities over time among survey periods. This approach serves to mitigate the impact of migration on our analysis (MacKenzie et al., 2018). To assess the adequacy of our models and the impacts of violating underlying assumptions, we conducted a posterior predictive check (PPC) as a goodness of fit test for all models using a χ^2^ fit statistic, calculated with the *spOccupancy* R package (Doser et al., 2022). Our analysis revealed no indication of a lack of fit in any of the seasonal models (PPC: winter = 0.39, spring = 0.41, summer = 0.42, fall = 0.40). If potential biases persist, we believe that these should not significantly distort comparisons between different sites or compromise our evaluation of how site characteristics interact with species behaviors, given the close proximity of our study sites, we reasonably anticipate that migratory birds' departure and arrival times will exhibit similar trends across the various locations.

Multispecies occupancy models assume that the set of species modeled is composed of species that will respond similarly, but not identically, to environmental variables (Dorazio et al., 2010; Kéry & Royle, 2016, 667). The models allow for heterogeneity in species responses to environmental conditions for both detectability and occupancy, including the direction of response. We address the assumption of ecological similarity by reducing the set of species modeled to represent those with similar resource use, dependence on nearby water bodies, and similar home ranges and body sizes. Based on these criteria, we included passerines (*Passeriformes*), hummingbirds (*Apodiformes*), doves (*Columbiformes*), and woodpeckers (*Piciformes*) (*n* = 80 species; see Table S3 in Appendix S1 for species list, scientific names, and the seasons in which species were detected). Nocturnal species, shorebirds, waterfowl, waterbirds, game birds, and raptors were excluded from the analysis. We assume that given the heterogeneity allowed by the model, species with different migratory patterns and species with varying urban tolerances can be modeled jointly. For the set of modeled species, we define the “community” as a set of species occurring in the same place at the same time (Ricklefs, 2008).

Model predictors of avian occupancy were fit to evaluate processes at the local scale (canopy cover, proportion native trees, impervious cover, total shrub cover, native shrub cover, and vertical complexity), neighborhood (matrix canopy cover and matrix impervious cover), and landscape scale (distance to baylands, distance to stream; Table S2 in Appendix S1). The pairwise Pearson correlation coefficient for all included environmental variables was <|0.6|. Detection was modeled as a function of Julian day and its quadratic, time after sunrise (in minutes), and the ambient noise at the site during the survey, measured by a phone app sound meter and external microphone (in *L*_eq_).

Field encounters of species *i* at site *j* during primary sampling period *k* and secondary period *t*, denoted as *y*_*ijkt*_ (*y* = 1 if the species was encountered and *y* = 0 otherwise), were assumed to be the result of the imperfect detection of a species' true incidence at a site: *z*_*ijk*_ (*z* = 1 if the species occurred at the site, and *z* = 0 otherwise). The incidence of a species was modeled as conditional on the probability that a species occupies that site ($\psi_{\mathrm{ijk}}$):

$$z_{\mathrm{ijk}}|\psi_{\mathrm{ijk}}\sim \text{Bernoulli}\;\left(\psi_{\mathrm{ijk}}\right)$$

whereas the field encounters were the probability of detecting that species during a survey, $p_{\text{ijkt}}$, given its incidence at a site:

$$y_{\text{ijkt}}|z_{\mathrm{ijk}},p_{\text{ijkt}}\sim \text{Bernoulli}\;\left(z_{\mathrm{ijk}}\times p_{\text{ijkt}}\right)$$

Occupancy and detection probabilities were modeled as linear combinations of covariates along with an intercept using a logit‐link transformation:

$$\text{logit}\left(\psi_{\mathrm{ijk}}\right)=\alpha {Ο}_{i}+\alpha_{i}{Χ}_{j}$$

$$\text{logit}\left(p_{\text{ijkt}}\right)=\beta {Ο}_{i}+\beta_{i}{Χ}_{\mathrm{jkt}}$$

Species‐specific intercept and covariate values were modeled as normally distributed governed by community‐level hyperparameters, for example:

$$\alpha {Ο}_{i}=\text{Normal}\left(\mu_{\mathrm{\alpha Ο},\text{community}},\tau_{\mathrm{\alpha Ο},\text{community}}\right)$$

All intercepts and covariates were drawn from separate distributions and followed this same form. The community‐level means and precisions were assigned the following priors:

$$\mu_{\mathrm{\alpha Ο},\text{community}}\sim \text{Normal}\left(0,\sigma^{2}=2.72\right)$$

$$\tau_{\mathrm{\alpha Ο},\text{community}}\sim \text{Inverse Gamma}\left(0.1,0.1\right)$$

which translates to a relatively uniform prior when transformed on the probability scale (0, 1). Research on prior selection has demonstrated this an appropriate specification for relatively non‐informative priors for occupancy models (Broms et al., 2016; Northrup & Gerber, 2018). Models were fit using Markov chain Monte Carlo methods as implemented by the *spOccupancy* package in R which employs Pólya‐Gamma data augmentation for efficient sampling (Doser et al., 2022). 13,200 samples of the posterior were generated for each model (three chains, 54,000 iterations, 10,000 burn‐in, and a thinning of 10). Pre‐ and post‐processing of data was done in R (R Core Team, 2020). Chains were considered converged if the Gelman‐Rubin statistic was <1.1 for all stochastic nodes (Gelman et al., 2014). Significance throughout the analyses is defined as values having non‐overlapping 95% highest‐density interval (HDI) when comparing numbers, or a 95% HDI that is non‐overlapping with zero when evaluating the significance of covariate values. Species‐specific responses to environmental variables were considered strong when their 75% HDIs did not overlap with zero. Code and spatially anonymized data in Iknayan et al. (published on acceptance). The community‐level distributions for covariates provide inferences of the drivers of overall community response, whereas species‐specific values can help investigate the responses of individual species that underlie the community response.

Total richness and richness of a subset of the community are presented throughout as the number of species potentially gained or lost by management actions, which likely provides a more intuitive summary of outcomes for landholders than occupancy probability values. One of the notable advantages of the Bayesian multispecies occupancy model is that it returns the estimation of every species' presence or absence at each survey site, represented as a binary incidence matrix (*z*_*ijk*_), as a fundamental output. In this matrix, zeros signify estimated absence at a site, and ones indicate estimated presence. This estimation is returned for every sample of the posterior distribution, making it straightforward to link error to the estimated presence or absence of each species. This feature enables the easy estimation of species richness within any chosen subset of the community by simply summing the incidence values from the resulting posterior distribution. Furthermore, the Bayesian framework extends this flexibility to making predictions. At any given value for a predicted variable, the model returns estimated incidence values for each species across the entire posterior distribution. As a result, errors in estimation can be directly associated with these predicted incidence values and for any incidence‐derived community‐level index such as richness. Throughout predictions were generated using the R function *spOccupancy::predict.tMsPGOcc*.

### 2.6 Predicting the relative contribution of spatial scales to avian richness

We made predictions about the relative contribution of local‐, neighborhood‐, and landscape scales to differences in annual bird species richness by simulating data for hypothetical sites under assumed “good” and “poor” quality conditions at each scale, based on the significant covariates returned by the community occupancy models in any season. At each spatial scale, we set the significant variables having positive or negative associations with community occupancy to their maximal or minimal values, respectively, and predicted annual richness at these hypothetical sites with different combinations of “good” or “poor” conditions at each scale. Our hypothetically “good” conditions at the local, neighborhood, and landscape scale were set to have the maximum canopy cover and minimum impervious cover at the local and neighborhood scale and the minimum distance from the Bay and streams source at the landscape scale (i.e., “good” conditions based on significant model coefficients associated with higher richness). All other environmental variables were held to their average values. Hypothetically “poor” sites were set to opposite conditions at those scales. We evaluated combinations of “good” and “poor” conditions at different scales to evaluate the impacts of different actions across scales.

### 2.7 Bird urban‐tolerance score

To evaluate whether site characteristics benefited more urban‐tolerant or more urban‐sensitive avian species, we used species‐level urban‐tolerance scores provided by Callaghan et al., 2021. Callaghan et al.'s analysis quantifies species‐specific responses to urbanization in the Western Hemisphere by integrating citizen science observations from eBird with urbanization intensity as measured by night‐time light intensity (Elvidge et al., 2017). We averaged Callaghan et al.'s monthly, species‐specific urban tolerance scores for the seasons in which a species was detected in the study area. Species scores were then classified into “urban sensitive” (0.33 quantile of the community‐level distribution of scores), “urban neutral” (0.33 to 0.66 interquartile range), and “urban tolerant” (0.66 to 1.00 interquartile range). The dataset covers all but three species in our analysis: golden‐crowned sparrow, cliff swallow, and willow flycatcher. Given the lack of data on these species, they were excluded from our assessment of urban tolerance. Previous work has found that continental measures of urban tolerance align with local measures of urban tolerance (Callaghan et al., 2020).

### 2.8 Pollinator taxonomic richness

Pollinator surveys did not include within‐season resurveys, preventing the use of occupancy models and the correction of potential variation in pollinator detectability. In light of these limitations, we assumed pooled raw pollinator taxonomic richness values from the surveys could provide an index to evaluate site characteristics' effects on pollinator diversity. Only native or potentially native taxa were included in the analysis and nonnative pollinators were excluded from analysis: *Apis* sp. and *Pieris rapae* (Table S1 in Appendix S1). Taxa with potentially invasive species were evaluated based on known ranges of invasive species (Droege, 2021): *Hylaeus* sp., *Megachile* sp., *Polistes* sp., *Vespula* sp. These taxa were included in the final analysis because they occurred in low numbers and models including and excluding these taxa returned the same significant covariates and similar covariate values. The same environmental variables used in the avian multi‐species occupancy model were included as predictors of pollinator richness using a Poisson linear model (GLM) with a variance of ϕ×μ, where μ is the mean and ϕ is the dispersion. Models were fit using the *glm()* function in R. A standard Poisson model was appropriate given that no overdispersion was detected in the standard errors.

### 2.9 Assessment of spatial autocorrelation

As site selection was restricted to participating landowners, we assessed potential spatial autocorrelation that could have resulted from the non‐random placement of sampling locations. For birds, we analyzed residual spatial autocorrelation using methods explicitly designed for occupancy models presented by Wright et al. (2019). Latent occupancy residuals for each species and site were obtained by subtracting the probability of occupancy ($\psi_{\mathrm{ijk}}$) from the incidence ($z_{\mathrm{ijk}}$) across the entire posterior distribution. These residuals may be more appropriately termed “discrepancies” as incidence is partially latent. We assessed spatial autocorrelation for every species in each model using Moran's I at 500 m distance bands from 0 to 5 km. Band distance was chosen following guidelines by Fletcher and Fortin (2018) to allow for a sufficient number of pairs for comparison in each distance band (>20 pairs) and capped at 5 km to avoid boundary effects. We found limited evidence of spatial autocorrelation in our avian models; only 0.34% (*n* = 7) of our species‐season pairs in any distance class in any season had 95% HDIs non‐overlapping zero (Figure S4 in Appendix S1). This overlap did not show a pattern with distance or seasonality, and there was no spatial autocorrelation evident for sites separated by <1 km. For pollinators, we calculated Moran's I using the R package *pgirmess::correlog*, using the Sturges method to compute the optimal number of distance classes (Giraudoux, 2023). There was no significant spatial autocorrelation evident for sites <5 km apart.

## 3 RESULTS

### 3.1 Birds

Our surveys recorded 10,832 encounters of 80 avian species across four seasons (first winter: 2564 encounters; spring: 2432; summer: 2238; fall: 1755; second winter: 1838; Table S3 and Figure S2 in Appendix S1). Average avian occupancy was highest in the summer (mean: 0.54, 95% HDI: 0.49 to 0.60; Figure S1 in Appendix S1) and lowest in the winter (mean: 0.45, 95% HDI: 0.42 to 0.49). Though average avian occupancy did not significantly vary across seasons, these results indicate that species were more widely distributed in the summer, and species were more patchily distributed in the winter. On average across the community, if a species was present at a site, the probability of it being detected through the course of all surveys at that site (*p* ^*★*^; MacKenzie et al., 2018) ranged from a mean of 0.72 to 0.84 (Table S4 in Appendix S1). Detectability was highest in the summer and lowest in the fall and spring, which aligns with expectations that birds are most detectable during the breeding season (Ehnes et al., 2018). However, these values did not vary significantly; potentially reflective of area searches being less dependent on auditory detections than point count methods (Pascoe et al., 2019). Detection probability coefficient values are presented in Table S6 in Appendix S1 and species detection probabilities by season are in Figure S3 in Appendix S1.

**FIGURE 2:**
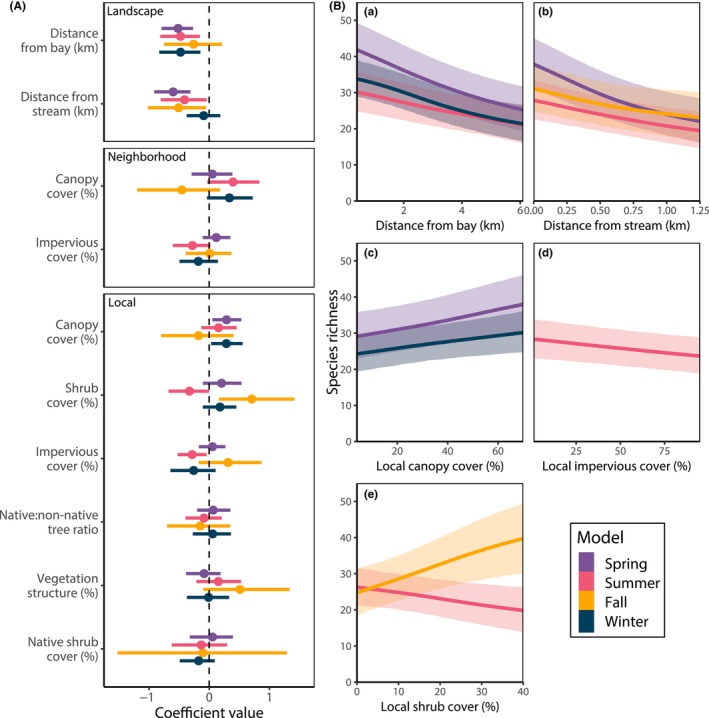
Coefficients and 95% HDI for all landscape, neighborhood, and local scale predictors in seasonal avian community occupancy models (A), and predicted change and 95% HDI in seasonal bird species richness across observed values of significant landscape (a, b) and local scale (c–e) predictors derived from seasonal multi‐species bird occupancy models (B). Legend colors refer to the same seasonal models in A and B. Full occupancy model summaries are in Table S5 in Appendix S1.

Environmental features at all three spatial scales influenced avian community occupancy. Both landscape‐scale features were strong predictors across three seasons, at least one local‐scale resource was a strong predictor in each season, and while HDIs included zero for all neighborhood‐scale features across seasons, two features at that scale appeared to have some influence on avian community occupancy in the summer or winter (Figure 2, Table S5 and Figure S2 in Appendix S1). At the landscape level, both the distance to streams and the Bay were significant predictors in three of four seasons (significance defined here as 95% HDIs not overlapping zero). Moving 0.5 km further from a stream resulted in the loss of more species (spring: 2.7 to 8.3; summer: 1.8 to 3.9; fall: 1.7 to 4.3) than moving the same distance from the Bay (spring: 0.8 to 1.8; summer: 0.7 to 0.9; winter: 0.6 to 1.4; Table S7 in Appendix S1). The significance of local‐scale predictors varied by season. Increasing local canopy cover was associated with increased species richness in both spring and winter (1.1 to 1.6 and 0.8 to 1.0 species gained per 10% increase in canopy cover, respectively), and higher local impervious cover was associated with decreased species richness in the summer (0.4 to 0.6 species lost per 10% increase in impervious cover). Increased local shrub cover had opposing effects as it was linked with decreasing species richness in the summer (1.2 to 1.9 species lost per 10% increase in cover) and increasing richness in the fall (2.5 to 4.1 species gained per 10% increase).

For these multi‐species occupancy models, significant predictors of species richness arose from similar responses by different species in the bird community. For example, the relationship of different species to proximity to the Bay was nearly uniform: spring, summer, and winter occupancy of 35% to 52% of species decreased as distances between sites and the Bay increased, while 0% to 5% of species' occupancy increased. Occupancy of 18% to 42% of species decreased as distances to urban streams increased in spring, summer, and fall, while 0%–4% of species were expected to increase in occupancy. 19% and 25%of species in the community responded positively to increases in local canopy cover in the spring and winter, respectively, and 0% and 1% of species responded negatively. Conversely, the occupancy of different groups of species showed opposing responses to neighborhood‐scale canopy cover during some seasons. For example in spring, summer, and fall, a subset of species within the community responded negatively (15%, 11%, and 28%, respectively) while a complement responded positively to increases in neighborhood‐scale canopy cover (18%, 28%, and 13%, respectively; Table S5 in Appendix S1). The species that declined with increasing canopy cover were for the most part habitat generalist species with a few exceptions: black phoebe (water‐associated; scientific names provided in Table S3 in Appendix S1), fox sparrow, and California towhee (shrub‐associated), and Bullock's oriole (forest‐ and parkland‐associated). In all seasons, the majority of species that responded positively to increasing canopy cover were forest‐associated species with the remainder being generalists.

**FIGURE 3:**
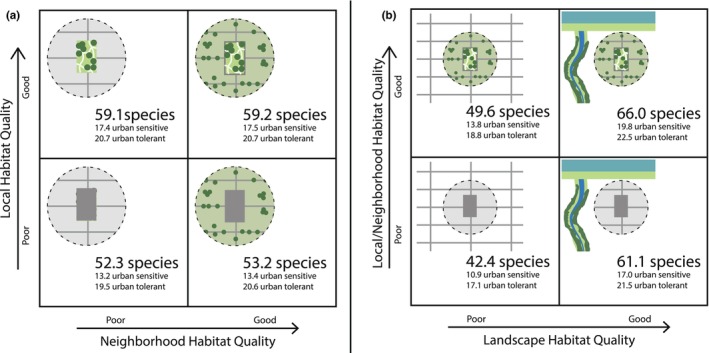
Predicted total annual bird species richness at sites under conditions that we assumed to represent “good” and “poor” quality sites at each scale, based on covariates of strong and consistent effect returned by the multi‐species occupancy model (Table S5 in Appendix S1). Covariates used were distance to bay and distance to stream (landscape scale) and canopy cover and impervious cover (local and neighborhood scale). At each spatial scale, we set covariates in any season to their maximal or minimal values and predicted annual species richness for all species, urban sensitive species, and urban tolerant species (Table S3 in Appendix S1) at these hypothetical sites.

Our examination of the relative contributions of natural features at three spatial scales on community occupancy revealed that predicted annual richness was 83% of the total (66.0 of 80 species; 95% HDI: 60.0 to 71.6) when local, neighborhood, and landscape scale features were set to their highest quality values (i.e., closest to the Bay and stream, highest canopy cover, and lowest impervious cover for both the neighborhood and local scales; Figure 3). Nonetheless, conditions we manipulated to represent the poorest conditions at all three scales (i.e., farthest from the Bay and stream, lowest canopy, and highest impervious cover) still supported about half of the total avian richness present in the study area (42.4 of 80 species; 95% HDI: 34.6 to 50.5; Figure 3). Between‐scale manipulations revealed that high‐quality sites embedded in poor‐quality neighborhoods had 5.9 (95% HDI: −4.25 to 16.0) more predicted bird species than low‐quality sites embedded in high‐quality neighborhoods. Furthermore, sites of poor local and neighborhood scale quality in high‐quality landscapes were predicted to harbor 11.5 (HDI 95%: 1.47 to 21.4) more species than sites of high local and neighborhood scale quality in poor‐quality landscapes (Figure 3).

Thirteen species were predicted to be likely (>0.90 occupancy probability) at all sites regardless of quality: American crow, American goldfinch, Anna's hummingbird, brown‐headed cowbird, black phoebe, bushtit, California towhee, dark‐eyed junco, house sparrow, lesser goldfinch, mourning dove, violet green swallow, and yellow‐rumped warbler. All of these species were categorized as urban tolerant (*n* = 8) or urban neutral (*n* = 3), except for the dark‐eyed junco and violet green swallow (characterized as urban sensitive). At least one urban sensitive species was detected at each site. Based on simulations, the lowest number of urban sensitive species (average of 10.9 species, 95% HDI: 6.9 to 15.1) were predicted at sites with the poorest conditions, whereas a comparable number of urban sensitive (19.8 species, 95% HDI: 15.9 to 23.4) versus urban tolerant species (22.5 species, 95% HDI: 19.9 to 24.9) were predicted at sites with the highest quality conditions (Figure 3). Occupancy of urban sensitive species was the most responsive to landscape‐level shifts in quality (predicted increase of 6.2 species (95% HDI: 1.06 to 11.2) in highest quality vs. poor quality landscapes regardless of the local/neighborhood scale conditions), followed by local scale conditions (predicted increase in 4.0 (95% HDI: −1.1 to 9.1) and 4.3 (95% HDI: −0.7 to 9.3) species in locally good vs. locally poor conditions under poor and good quality neighborhood conditions, respectively). Neighborhood scale conditions appeared to have little effect on urban sensitive species (0.3 [95% HDI: −6.2 to 5.7] and 0.0 [95% HDI: −1.1 to 9.1] shift in species in poor vs. good neighborhood conditions, under poor and good quality local conditions, respectively). Urban tolerant species occupancy was highest when conditions were set to their best versus poorest at all three scales, with the number of predicted urban tolerant species increasing similarly when landscape conditions improved under poor (21.5 [95% HDI: 18.6 to 24.2] vs. 17.1 [95% HDI: 13.2 to 21.0] species) versus good (22.5 [95% HDI: 19.9 to 24.9] vs. 18.8 [95% HDI: 15.7 to 21.8] species) local/neighborhood conditions (Figure 3).

### 3.2 Pollinators

We encountered 331 pollinators interacting with floral reproductive parts during surveys. Pollinator taxonomic richness consisted of 42 taxa (grouped either into species or genera), including 40 identified genera of the orders *Lepidoptera* (14 genera, 12 identified species), *Hymenoptera* (23 genera), and *Diptera* (3 genera; Table S1 in Appendix S1). All pollinators except *Apis* sp. and *Pieris rapae* were native to North America. Similarly to birds, shifts in seasons and spatial scales also influenced patterns in pollinator richness (Table 1). In spring, only local‐scale covariates were important predictors of pollinator richness, when decreasing proportions of both tree canopy cover and impervious cover and higher native tree to non‐native tree ratios were associated with increasing indices of richness (Table 1). In both the early and late summer, variation in pollinator richness was best explained by the landscape‐scale measure of distance from the San Francisco Bay, when sites closer to the bay had higher pollinator richness than those farther away (Table 1).

**TABLE 1:** Results of seasonal pollinator richness models.

	Spring model			Early summer model			Late summer model		
	β	SE	95% CI	β	SE	95% CI	β	SE	95% CI
Intercept	1.06	0.11	0.84 to 1.26	1.10	0.11	0.88 to 1.30	0.76	0.21	0.34 to 1.15
Landscape									
Dist. from the bay (km)	−0.12	0.16	−0.45 to 0.20	**−0.37**	**0.18**	**−0.72** to **−0.03**	**−0.90**	**0.41**	**−1.70** to **−0.10**
Dist. from stream (km)	0.07	0.13	−0.19 to 0.33	0.07	0.16	−0.24 to 0.38	0.18	0.21	−0.22 to 0.61
Neighborhood									
Canopy cover (%)	0.07	0.15	−0.23 to 0.37	0.09	0.17	−0.25 to 0.44	0.61	0.40	−0.17 to 1.38
Impervious cover (%)	0.02	0.16	−0.29 to 0.35	0.14	0.16	−0.16 to 0.47	−0.06	0.24	−0.52 to 0.43
Local									
Canopy cover (%)	**−0.28**	**0.12**	**−0.51** to **−0.05**	−0.10	0.12	−0.34 to 0.13	−0.03	0.15	−0.33 to 0.27
Shrub cover (%)	−0.11	0.14	−0.38 to 0.16	−0.09	0.16	−0.41 to 0.24	0.15	0.22	−0.28 to 0.60
Impervious cover (%)	**−0.24**	**0.11**	**−0.44** to **−0.01**	−0.19	0.15	−0.48 to 0.10	−0.15	0.18	−0.51 to 0.21
Vegetation structure (%)	0.10	0.11	−0.14 to 0.31	0.02	0.12	−0.23 to 0.24	−0.04	0.13	−0.32 to 0.21
Native shrub cover (%)	0.04	0.11	−0.19 to 0.25	0.08	0.14	−0.21 to 0.36	−0.12	0.18	−0.49 to 0.22
Native: non‐native tree ratio	*0.21*	*0.10*	*0.00 to 0.41*	0.02	0.13	−0.25 to 0.25	−0.03	0.15	−0.35 to 0.24

*Note*: Poisson linear regression coefficients (β), standard errors (SE), and 95% confidence intervals. Bolded coefficients are those for which 95% confidence intervals did not include zero and italicized coefficients are those for which intervals include zero but there is a tendency toward one direction of effect.

## 4 DISCUSSION

Integration of natural elements into the urban landscape creates the opportunity for a “win‐win ecology” for wildlife and human urban residents (Rosenzweig, 2003). Here we focused on patterns in bird community occupancy and pollinator richness across local‐scale sites with varying cover and complexity of natural vegetation, embedded in neighborhoods of variable proportions of tree canopy and impervious surface, within a highly developed landscape with variously‐spaced water features. Spatial, seasonal, and taxonomic scope were all important for understanding how birds and pollinators occupied these urban sites. Landscape‐, local‐, and to some extent neighborhood‐scale conditions influenced the avian community, while landscape‐ and local‐scale characteristics were the best predictors of the pollinator community. The scale‐defined conditions that were important for both taxa, however, varied by season (Figure 2). If surveys had been limited to only one season—for example, spring for pollinators and summer breeding season for birds—the importance of landscape‐level resources for pollinators and additional site‐scale resources for birds would have been overlooked (Figure 2, Table 1). Because we used the same measure of environmental features across seasons, however, it is difficult to determine the degree to which these effects are attributed to either the changing animal compositions between seasons or to seasonal changes in environmental features (which we did not measure).

Our evaluation of scale—regardless of season—suggests that a site's position among natural features in the wider landscape has a stronger effect on avian species richness than site‐ and neighborhood‐scale conditions (Figure 3b). Yet, even when a site is distant from regionally important resources such as stream corridors and large estuarine water bodies—a feature over which managers tend to not have control—42.4 species (95% HDI: 34.6 to 50.5) are expected to occupy a low‐quality site in a low‐quality neighborhood and could be improved to 49.6 species (95% HDI: 42.4 to 56.8) if impervious surfaces are replaced with canopy cover. Additionally, bird species sensitive to urbanization appeared to benefit most from a combination of good conditions at the site and landscape scale (Figure 3), implying that improving conditions within the urban landscape (e.g., increasing tree canopy) could have an impact on which species are able to tolerate living in cities.

Our work largely corroborates existing research on the natural features in urban areas that improve community measures of birds and pollinators but improves our understanding of the scales and seasons at which these features appear to have their greatest effect. Our work suggests that protecting sites near regional water bodies and reducing impervious surfaces at the local scale will increase avian occupancy and species richness and pollinator richness in urban areas. While managing for higher local and neighborhood scale tree canopy cover will likely promote overall avian diversity, providing localized spaces free from the tree canopy and impervious surface cover (thus allowing for better flower growth in the open sun) will likely promote pollinator richness.

### 4.1 The full annual cycle matters

We found that the predictors of bird and pollinator richness varied depending on season; thus to maximize urban landscapes for birds and pollinators, all seasons should be considered. Although the strength of landscape‐scale effects on both avian and pollinator richness varied seasonally, the direction of effect did not (Figures 2 and 3, Table 1 and Table S5 in Appendix S1). There were, however, seasonal contradictions in the direction of local and neighborhood effects on bird occupancy and richness (Figure 2, Table S5 in Appendix S1). The most striking of these was the negative effect of local shrub cover in the summer and the positive effect in other seasons, most strongly so in fall. Because the shrub species in the study area are mostly evergreen, and we used summer measures of shrub cover for models in other seasons, we suspect this result reflects the shifting bird community composition between seasons. Bird assemblages shift throughout the year, with some species present year‐round and others present on the scale of days or months during migration, breeding, or wintering (Marra et al., 2015). For example, two migratory species that occupy the study sites, white‐crowned and golden‐crowned sparrows, are not present in summer but form conspicuous social flocks among shrubby suburban habitats in late fall, winter, and early summer (Figure S2 in Appendix S1; Price, 1931, Robertson, 1957). Pollinator assemblages are similarly dynamic throughout the year (Olesen et al., 2008; Wojcik et al., 2008). Each seasonal assemblage has different needs from the landscape. For example, streams, tree canopy, and shrub cover were important for birds in spring or fall; features that may be acting as visual cues for migrating birds to locate stop‐over sites (van Riper et al., 2008). Site‐scale characteristics were more important for early emerging pollinators, and landscape‐scale characteristics were more important for later emerging pollinators, perhaps reflecting a shift toward the Bay as the summer progresses and the urban heat effect increases (Table 1). Current scientific knowledge about which features of urban areas are associated with avian community measures is mainly limited to the breeding season (Lepczyk et al., 2017)—a bias that permeates research across taxa (Marra et al., 2015). The relationship between features of urban greenspace across the full annual cycle of birds has been documented in only one other study we are aware of; La Sorte et al. (2020) also found the features that predicted avian diversity varied by season. Studies of wintering urban bird habitat selection are equally infrequent (Lepczyk et al., 2017). Seasonality of pollinators is better studied (Leong et al., 2016; Wenzel et al., 2020; Wojcik et al., 2008), as pollinator diversity is inextricably linked to the phenology of the plant and floral resources on which this taxon depends. Understanding which features are associated with components of biodiversity throughout the annual cycle allows for the conservation of multiple temporal communities in one geographic location (Faaborg et al., 2010; Lin et al., 2020), and can influence carry‐over effects that migrating animals take with them to different locations and physiological states (Marra et al., 2015).

Protecting and enhancing migratory habitat for birds in urban areas is particularly important. Birds are vulnerable during migration when mortality rates are higher than in other seasons (Klaassen et al., 2014; Sillett & Holmes, 2002). Further, Neotropical migrants are more likely to occur in urban areas during migration (Zuckerberg et al., 2016), and most priority stopover sites for migratory birds in North America are in human‐modified landscapes (Lin et al., 2020). Small patches of urban habitat, such as green roofs, can provide habitat for refueling (Matthews & Rodewald, 2010; Partridge & Clark, 2018). All sites in this study had at least one migratory species (spring mean: 1.8 species per site, 95% HDI: 0.8 to 3.1; fall mean: 1.39 species per site, 95% HDI: 0.7 to 2.4), which supports the recommendation that habitat for migratory species should be maintained throughout the urban matrix (La Sorte et al., 2014). Riparian areas are important stop‐over sites during migration (e.g., Cormier et al., 2013), including in urban systems (Pennington et al., 2008), and we found that distance from the nearest stream was significant during both spring and fall (Figure 2). This may indicate these are important refueling areas for migratory species in this region or that the open spaces associated with riparian corridors are acting as a visual cue for locating stopover habitat. Dense vegetation in the form of local tree canopy cover in the spring and local shrub cover in the fall was also associated with higher bird diversity during migration in our study. Dense vegetation can act as a visual cue for migrating species (van Riper et al., 2008), and higher canopy cover is known to attract a higher diversity of migratory birds in the spring (La Sorte et al., 2020). Conservation of stop‐over habitats for migratory birds is a critical step for protecting these species throughout their annual cycle.

### 4.2 Integrate management actions across scales

Our combined findings indicate that management actions should be integrated across spatial scales for maximum occupancy and taxonomic richness of birds and pollinators. Patterns in both birds and pollinator diversity are driven by features at both local and broader geographic scales (Lepczyk et al., 2017; Wenzel et al., 2020). In our study, features at the local and landscape scale were strong predictors of avian occupancy and richness and pollinator richness, while neighborhood‐scale predictors also influenced bird occupancy somewhat (Table 1, Figure 2). Our simulations predicted that a site with local and neighborhood habitat improvements had 16.4 fewer bird species if the surrounding landscape was poorer in quality. Similarly, bird species richness was limited by site conditions even if the neighborhood and landscape in which sites were embedded were of high quality (Figure 3a,b). These types of relationships between avian diversity and natural features at multiple scales are commonly found (e.g., Hennings & Edge, 2003; Lerman et al., 2021; Litteral & Shochat, 2017). Taken together, our results imply that integrating management and restoration actions at the local and neighborhood scale while minimizing impacts near regional high‐quality resources could work holistically to maximize bird and pollinator richness across the region. However, our results should empower stakeholders to improve conditions for urban wildlife at the scale over which they have control, as improvements at each scale were independently predicted to contribute to increased avian occupancy or pollinator richness (Figure 2, Table 1). Further, incremental habitat improvements likely contribute to increases in bird and pollinator occupancy r; predicted effects were mostly linear and did not indicate thresholds of effect across the values measured (Figure 2).

### 4.3 Minimize human impacts near regional resources

Urban areas are often perceived as disconnected from the surrounding natural environment (Spotswood et al., 2021); however, our results highlight the importance of proximity to regional natural resources, namely streams and baylands, to birds and pollinators. Water bodies in urban areas—even artificially constructed features such as stormwater detention basins (Blackwell et al., 2008)—can provide invaluable resources and can form biodiversity hotspots (Canedoli et al., 2018; Fernandez‐Juricic, 2000; Tilghman, 1987). Even though we selected for study species that were not dependent on the presence of water bodies (i.e., we excluded shorebirds, waterfowl, and waterbirds from our analyses), proximity to the San Francisco Bay influenced multi‐species occupancy in all seasons except the fall (Figure 2). Furthermore, distance from the Bay was a significant factor in the decrease of pollinator diversity in the early and late summer, which is a known landscape‐level factor that influences the diversity of pollinator gardens (Majewska & Altizer, 2020). Because human development is concentrated along coastlines, pressures from development may disproportionately threaten shoreline natural resources (Guetté et al., 2018). Thus, concerted efforts should be made to protect urban shorelines. Similarly, riparian sites in urban areas can harbor a disproportionate number of bird species compared to non‐riparian sites, and bird species richness tends to become more concentrated in riparian areas as surrounding development pressure increases (Bennett et al., 2014). This aligns with our model prediction that bird species richness could increase by as much as four species for every 500 m that a site is closer to an urban stream (Table S7 in Appendix S1). Building wide setbacks near creeks and creating channel enhancements and wide vegetated patches near creeks should increase regional bird diversity.

### 4.4 Small‐scale improvements matter

Though a site's location near‐to or far‐from regional resources had the strongest effects on avian richness, local‐scale quality was also important for avian richness in either poor or high‐quality landscapes, according to our model predictions (Figure 3b). At even the poorest quality sites, nestled deep in the developed urban matrix, we detected about half of the total avian annual richness. Our model predictions suggested that increasing canopy cover and reducing the impervious surface area at the local‐ and neighborhood scale could be associated with an additional 7.1 bird species (95% HDI: −3.7 to 18.0) throughout the year compared to the poor quality baseline and that the same local and neighborhood improvements in high‐quality landscapes could increase richness by 4.9 species (95% HDI: −3.6 to 13.4; Figure 3b). Several studies have found positive responses by birds to urban tree cover (Chong et al., 2019; Kalinowski & Johnson, 2010 and La Sorte et al., 2020), and loss of canopy cover can often have negative impacts on urban communities through increased exposure to predators (e.g., Evans et al., 2009). Increasing canopy cover was considered by Hennings and Edge (2003) to be the most valuable action for conserving native bird species in urban landscapes.

Despite the importance of canopy cover for birds, increasing local‐scale canopy cover negatively impacted early spring pollinator richness, representing the only environmental variable where the two taxa had conflicting responses (Figure 2 and Table 1). Further, no neighborhood‐scale features were significant predictors of pollinator richness (Table 1). These findings highlight that some aspects of local and neighborhood scale habitat enhancements will need to be managed differently to improve conditions for rt pollinators as well as birds. The decline in pollinator diversity with increasing canopy cover has been attributed to shading that limits the growth of floral resources (Farwig et al., 2009; Grundel et al., 2010; McCabe et al., 2019). Finally, the amount of impervious cover at the local scale was negatively associated with pollinator richness, which could reflect the loss of nesting habitat and food resources for these species when impervious cover is high (Wenzel et al., 2020). This filter appeared to be lifted for later season pollinators, however, which were more limited by a site's proximity to the Bay.

### 4.5 Urban sensitive versus urban tolerant species

It remains a question whether avian species richness is the best metric for determining the quality of a site (Hillebrand et al., 2018), particularly in urban areas where the most prevalent species may not be those of high conservation concern. In this study, however, a greater number of urban sensitive species (relative to urban tolerant species) were predicted to be gained at the best quality sites (Figure 3), indicating that the natural features at the scales we examined disproportionately benefited urban sensitive species. Only three species in our analysis are considered to be fully synanthropic: European starling, house sparrow, and rock pigeon. 76% of the species we observed, however, are known to at least occasionally exploit anthropogenic resources (Johnston, 2001). We did not find competing priorities in how urban tolerant and sensitive species responded to our predictors: higher‐quality sites either gained both urban tolerant and urban sensitive species (i.e., for local or landscape scale changes) or exhibited no change in these groups (i.e., for neighborhood scale changes; Figure 3).

### 4.6 Policy approaches to promoting urban wildlife

Creating a biodiverse city will require action at multiple scales through coordination among multiple stakeholders (Gaston et al., 2013; Goddard et al., 2010). There are many existing policy approaches to promote urban biodiversity: municipalities across North America have created ordinances to protect urban rivers, increase native landscaping, preserve urban trees, and promote dark skies, among other nature‐forward policy approaches (Brown, 2016). Enhancing biodiversity in urban settings necessitates considering the influence of seasonal factors in wildlife management plans. While recognizing the ecological significance of seasonality, it's important to acknowledge the practical challenges of implementing seasonal‐specific management actions. Understanding which actions might have varying effects in different seasons allows for informed decision‐making that incorporates trade‐offs into planning strategies.

Our results imply that both top‐down approaches that plan for biodiversity enhancements at the landscape‐scale as well as bottom‐up approaches that improve conditions at the local scale can work together to improve the occupancy and richness of birds and pollinators in cities. Actions at the landscape scale such as city‐wide biodiversity strategies, park master plans, and green infrastructure plans, can work to protect regionally important biodiversity resources such as large water bodies, large parks, and stream corridors. Wildlife zoning and development ordinances can help regulate where and how development occurs (Brown, 2016). Policy tools such as habitat overlay zones and requirements for wildlife‐friendly design near shorelines and streams could help lessen the impact of development. Transferable development rights can also help concentrate development in less sensitive areas. Progressive forest master plans can help cities reach and sustain canopy targets for municipally owned and managed trees at the neighborhood scale. Bottom‐up approaches such as outreach programs focused on individual property owners could help to improve conditions at local scales and incentive‐based structures such as tax credits can encourage this voluntary participation by private landowners. Our results suggest that local‐scale actions even in locations distant from regional resources can have a measurable effect on species richness, particularly if combined with city‐wide targets such as canopy cover goals that could simultaneously improve the neighborhood around sites alongside local‐scale actions.

### 4.7 Limitations of the study

The findings and interpretations of this study should be considered in light of three key limitations. First, we could not account for pollinator detectability as this portion of the study was not designed with repeated surveys. Second, vegetation data was not collected during three of the four seasons when bird and pollinator data was collected. Our model coefficients linking vegetation cover to bird occupancy and pollinator richness during each season were therefore based on the assumption that vegetation data collected during the summer months—when leaves were full and flowers were blooming—would be an adequate proxy for the other seasons during which leaves and herbaceous plants were in different stages of senescence. This likely biased winter models by using data that overestimated vegetation cover and structure. Studies examining species responses to vegetation characteristics during different seasons should prioritize collecting vegetation data during all seasons of the study. Third, though we collected data on floral resources during each pollinator survey, we were unable to include these important data in our analysis. Thus, our pollinator model results likely overestimate the positive or negative associations with the remaining variables.

## 5 CONCLUSION

Both abiotic and urban design factors affect birds and pollinators in urban areas. Evaluating the determinants of occupancy and richness through multi‐species occupancy models allows for the flexibility to quantify community‐level responses to management actions while also being able to parse out the responses of subsets of the community. Evaluating abiotic factors, such as distances from regional resources, can identify sites that have a higher propensity to harbor a higher number of species and are suitable targets for lowered or modified development intensity. Identification of important biotic factors, such as vegetation cover, can suggest targets for management actions. Here we show the capacity for birds and pollinators to occupy even highly developed landscapes. Generally, more available nature and resources result in a more biodiverse urban landscape; however, space is often limited in cities, thus restricting the total potential amount of greening that can be achieved. Nonetheless, even modest changes to increase canopy cover or reduce impervious cover can have large impacts, and over time such incremental changes can improve conditions for birds and pollinators.

## AUTHOR CONTRIBUTIONS

**Kelly J. Iknayan:** Formal analysis (lead); investigation (lead); project administration (lead); supervision (lead); validation (lead); visualization (lead); writing – original draft (lead); writing – review and editing (lead). **Sacha K. Heath:** Formal analysis (supporting); project administration (supporting); validation (supporting); visualization (equal); writing – review and editing (equal). **Scott B. Terrill:** Conceptualization (equal); data curation (equal); investigation (equal); methodology (equal); supervision (equal); writing – review and editing (supporting). **Daniel G. Wenny:** Data curation (equal); investigation (equal); methodology (equal); writing – review and editing (equal). **Stephanie Panlasigui:** Writing – original draft (supporting); writing – review and editing (equal). **Yiwei Wang:** Conceptualization (equal); investigation (equal); methodology (equal); writing – review and editing (equal). **Erin E. Beller:** Conceptualization (equal); resources (lead); writing – review and editing (supporting). **Erica N. Spotswood:** Formal analysis (supporting); project administration (lead); supervision (lead); writing – original draft (supporting); writing – review and editing (supporting).

## CONFLICT OF INTEREST STATEMENT

S. Heath was previously an Associate Editor for Ecology & Evolution; the remaining authors have no conflicts of interest to declare.

## Supporting information


Appendix S1:

## Data Availability

Data, code, and metadata are deposited at Iknayan et al. (2023) (https://doi.org/10.5061/dryad.0k6djhb68).
