# Supplementary material for: Patterns in bird and pollinator occupancy and richness in a mosaic of urban office parks across scales and seasons
Source: Ecol Evol. 2024 Mar 1;14(3):e10958. doi: 10.1002/ece3.10958 (PMC10905236; doi:10.1002/ece3.10958)
Supplement: Supplementary file 1 — Appendix S1: [file ECE3-14-e10958-s001.docx]

## Appendix 1: Table 1. Pollinator taxa observed and included in analyses. Dipterans were identified to family, Hymenopterans were identified to genera, and Lepidopterans to species, except for Hesperia sp., Hylephila sp., and Phyciodes sp. Taxa non-native to North American indicated.

| Taxonomic Group | Order | Family | Nonnative |
| --- | --- | --- | --- |
| *Bombyliidae gn.* | Diptera | Bombyliidae |  |
| *Muscidae gn.* | Diptera | Muscidae |  |
| *Syrphidae gn.* | Diptera | Syrphidae |  |
| *Andrena sp.* | Hymenoptera | Andrenidae |  |
| *Panurginus sp.* | Hymenoptera | Andrenidae |  |
| *Perdita sp.* | Hymenoptera | Andrenidae |  |
| *Pseudopanurgus sp.* | Hymenoptera | Andrenidae |  |
| *Anthophora sp.* | Hymenoptera | Apidae |  |
| *Anthophorula sp.* | Hymenoptera | Apidae |  |
| *Apis sp.* | Hymenoptera | Apidae | Yes |
| *Bombus sp.* | Hymenoptera | Apidae |  |
| *Ceratina sp.* | Hymenoptera | Apidae |  |
| *Melissodes sp.* | Hymenoptera | Apidae |  |
| *Nomada sp.* | Hymenoptera | Apidae |  |
| *Xylocopa sp.* | Hymenoptera | Apidae |  |
| *Colletes sp.* | Hymenoptera | Colletidae |  |
| *Hylaeus sp.* | Hymenoptera | Colletidae |  |
| *Agapostemon sp.* | Hymenoptera | Halictidae |  |
| *Augochlora sp.* | Hymenoptera | Halictidae |  |
| *Halictus sp.* | Hymenoptera | Halictidae |  |
| *Lasioglossum sp.* | Hymenoptera | Halictidae |  |
| *Sphecodes sp.* | Hymenoptera | Halictidae |  |
| *Hoplitis sp.* | Hymenoptera | Megachilidae |  |
| *Megachile sp.* | Hymenoptera | Megachilidae |  |
| *Osmia sp.* | Hymenoptera | Megachilidae |  |
| *Polistes sp.* | Hymenoptera | Vespidae |  |
| *Vespula sp.* | Hymenoptera | Vespidae |  |
| *Erynnis tristis* | Lepidoptera | Hesperiidae |  |
| *Hesperia sp.* | Lepidoptera | Hesperiidae |  |
| *Hylephila phyleus* | Lepidoptera | Hesperiidae |  |
| *Hylephila sp.* | Lepidoptera | Hesperiidae |  |
| *Poanes melane* | Lepidoptera | Hesperiidae |  |
| *Pyrgus communis* | Lepidoptera | Hesperiidae |  |
| *Plebejus acmon* | Lepidoptera | Lycaenidae |  |
| *Adelpha californica* | Lepidoptera | Nymphalidae |  |
| *Danaus plexippus* | Lepidoptera | Nymphalidae |  |
| *Junonia coenia* | Lepidoptera | Nymphalidae |  |
| *Phyciodes sp.* | Lepidoptera | Nymphalidae |  |
| *Vanessa cardui* | Lepidoptera | Nymphalidae |  |
| *Papilio rutulus* | Lepidoptera | Papilionidae |  |
| *Colias eurytheme* | Lepidoptera | Pieridae |  |
| *Pieris rapae* | Lepidoptera | Pieridae | Yes |

##

## Appendix 1: Table 2. Summary statistics for each environmental variable for all sites (n = 45).

|  | | Covariate | Mean | Median | Minimum | Maximum |
| --- | --- | --- | --- | --- | --- | --- |
| Local | | |  |  |  |  |
|  | Canopy cover (%) | | 27.1 | 25.0 | 0.0 | 70.0 |
|  | Impervious cover (%) | | 65.2 | 80.0 | 1.0 | 95.0 |
|  | Native:non-native tree ratio | | 7.5 | 0.0 | 0.0 | 88.0 |
|  | Shrub cover (%) | | 8.4 | 5.0 | 0.0 | 40.0 |
|  | Native shrub cover (%) | | 3.7 | 0.0 | 0.0 | 47.7 |
|  | Vertical vegetation complexity (%) | | 9.0 | 4.9 | 0.0 | 59.7 |
| Neighborhood | | |  |  |  |  |
|  | Canopy cover (%) | | 14.2 | 15.1 | 1.6 | 24.4 |
|  | Impervious cover (%) | | 51.2 | 52.5 | 11.4 | 74.5 |
| Landscape | | |  |  |  |  |
|  | Distance from bay (km) | | 3.3 | 2.9 | 0.4 | 6.1 |
|  | Distance from stream (km) | | 0.4 | 0.3 | 0.0 | 1.3 |

##

## Appendix 1: Table 3. Avian species included in the analyses along with their urban tolerance category. Seasons in which the species were modeled are indicated (W = wintering season; Sp = spring migration; Sum = summer; F = fall migration; All = all seasons). Four-letter codes are from the Institute for Bird Populations (Pyle and DeSante 2003). Species urban tolerances were based on species urban scores derived from Callaghan et al (2021): urban sensitive (species with scores in the 0.33 quantile of the community-level urban tolerance distribution of scores), urban neutral (0.33 to 0.66 interquartile range), and urban tolerant (0.66 to 1.00 interquartile range).

| Code | Common Name | Scientific Binomial | Family | Seasons | Urban tolerance |
| --- | --- | --- | --- | --- | --- |
| ROPI | Rock Pigeon | *Columba livia* | *Columbidae* | All | Tolerant |
| EUCD | Eurasian Collared-Dove | *Streptopelia decaocto* | *Columbidae* | All | Neutral |
| MODO | Mourning Dove | *Zenaida macroura* | *Columbidae* | All | Tolerant |
| VASW | Vaux's Swift | *Chaetura vauxi* | *Apodidae* | Sp,S,F | Neutral |
| WTSW | White-throated Swift | *Aeronautes saxatalis* | *Apodidae* | All | Tolerant |
| ANHU | Anna's Hummingbird | *Calypte anna* | *Trochilidae* | All | Tolerant |
| RBSA | Red-breasted Sapsucker | *Sphyrapicus ruber* | *Picidae* | W | Neutral |
| DOWO | Downy Woodpecker | *Dryobates pubescens* | *Picidae* | All | Tolerant |
| NUWO | Nuttall's Woodpecker | *Dryobates nuttallii* | *Picidae* | All | Tolerant |
| HAWO | Hairy Woodpecker | *Dryobates villosus* | *Picidae* | W | Sensitive |
| NOFL | Northern Flicker | *Colaptes auratus* | *Picidae* | W,Sp,F | Neutral |
| WEWP | Western Wood-Pewee | *Contopus sordidulus* | *Tyrannidae* | F | Sensitive |
| WIFL | Willow Flycatcher | *Empidonax traillii* | *Tyrannidae* | F | Neutral |
| PSFL | Pacific-slope Flycatcher | *Empidonax difficilis* | *Tyrannidae* | Sp,F | Tolerant |
| BLPH | Black Phoebe | *Sayornis nigricans* | *Tyrannidae* | All | Tolerant |
| SAPH | Say's Phoebe | *Sayornis saya* | *Tyrannidae* | W | Neutral |
| HUVI | Hutton's Vireo | *Vireo huttoni* | *Vireonidae* | W,Sp,F | Sensitive |
| CAVI | Cassin's Vireo | *Vireo cassinii* | *Vireonidae* | W,Sp | Sensitive |
| WAVI | Warbling Vireo | *Vireo gilvus* | *Vireonidae* | Sp,S,F | Neutral |
| CASJ | California Scrub-Jay | *Aphelocoma californica* | *Corvidae* | All | Tolerant |
| AMCR | American Crow | *Corvus brachyrhynchos* | *Corvidae* | All | Neutral |
| CORA | Common Raven | *Corvus corax* | *Corvidae* | All | Sensitive |
| TRES | Tree Swallow | *Tachycineta bicolor* | *Hirundinidae* | Sp | Neutral |
| VGSW | Violet-green Swallow | *Tachycineta thalassina* | *Hirundinidae* | Sp,S,F | Sensitive |
| NRWS | Northern Rough-winged Swallow | *Stelgidopteryx serripennis* | *Hirundinidae* | Sp,S,F | Neutral |
| CLSW | Cliff Swallow | *Petrochelidon pyrrhonota* | *Hirundinidae* | Sp,S,F | Neutral |
| BARS | Barn Swallow | *Hirundo rustica* | *Hirundinidae* | Sp,S,F | Sensitive |
| CBCH | Chestnut-backed Chickadee | *Poecile rufescens* | *Paridae* | All | Neutral |
| OATI | Oak Titmouse | *Baeolophus inornatus* | *Paridae* | All | Neutral |
| BUSH | Bushtit | *Psaltriparus minimus* | *Aegithalidae* | All | Tolerant |
| RBNU | Red-breasted Nuthatch | *Sitta canadensis* | *Sittidae* | W,Sp | Sensitive |
| WBNU | White-breasted Nuthatch | *Sitta carolinensis* | *Sittidae* | W,Sp,S | Neutral |
| BRCR | Brown Creeper | *Certhia americana* | *Certhiidae* | All | Sensitive |
| HOWR | House Wren | *Troglodytes aedon* | *Troglodytidae* | W,F | Neutral |
| MAWR | Marsh Wren | *Cistothorus palustris* | *Troglodytidae* | W,Sp,F | Sensitive |
| BEWR | Bewick's Wren | *Thryomanes bewickii* | *Troglodytidae* | All | Neutral |
| BGGN | Blue-gray Gnatcatcher | *Polioptila caerulea* | *Polio Ptilidae* | W | Tolerant |
| RCKI | Ruby-crowned Kinglet | *Regulus calendula* | *Regulidae* | W,Sp,F | Neutral |
| WEBL | Western Bluebird | *Sialia mexicana* | *Turdidae* | All | Sensitive |
| SWTH | Swainson's Thrush | *Catharus ustulatus* | *Turdidae* | F | Tolerant |
| HETH | Hermit Thrush | *Catharus guttatus* | *Turdidae* | All | Neutral |
| AMRO | American Robin | *Turdus migratorius* | *Turdidae* | All | Tolerant |
| VATH | Varied Thrush | *Ixoreus naevius* | *Turdidae* | W | Sensitive |
| NOMO | Northern Mockingbird | *Mimus polyglottos* | *Mimidae* | All | Tolerant |
| EUST | European Starling | *Sturnus vulgaris* | *Sturnidae* | All | Tolerant |
| CEDW | Cedar Waxwing | *Bombycilla cedrorum* | *Bombycillidae* | W,Sp,S | Tolerant |
| HOSP | House Sparrow | *Passer domesticus* | *Passeridae* | All | Tolerant |
| HOFI | House Finch | *Haemorhous mexicanus* | *Fringillidae* | All | Tolerant |
| PUFI | Purple Finch | *Haemorhous purpureus* | *Fringillidae* | W,Sp | Sensitive |
| PISI | Pine Siskin | *Spinus pinus* | *Fringillidae* | W,Sp | Sensitive |
| LEGO | Lesser Goldfinch | *Spinus psaltria* | *Fringillidae* | All | Tolerant |
| AMGO | American Goldfinch | *Spinus tristis* | *Fringillidae* | All | Neutral |
| SPTO | Spotted Towhee | *Pipilo maculatus* | *Emberizidae* | W,S,F | Sensitive |
| CALT | California Towhee | *Melozone crissalis* | *Emberizidae* | All | Tolerant |
| CHSP | Chipping Sparrow | *Spizella passerina* | *Emberizidae* | Sp | Neutral |
| SAVS | Savannah Sparrow | *Passerculus sandwichensis* | *Emberizidae* | W,F | Sensitive |
| FOSP | Fox Sparrow | *Passerella iliaca* | *Emberizidae* | W,Sp,F | Neutral |
| SOSP | Song Sparrow | *Melospiza melodia* | *Emberizidae* | All | Neutral |
| LISP | Lincoln's Sparrow | *Melospiza lincolnii* | *Emberizidae* | W,Sp,F | Neutral |
| WTSP | White-throated Sparrow | *Zonotrichia albicollis* | *Emberizidae* | W,Sp | Tolerant |
| WCSP | White-crowned Sparrow | *Zonotrichia leucophrys* | *Emberizidae* | W,Sp,F | Neutral |
| GCSP | Golden-crowned Sparrow | *Zonotrichia atricapilla* | *Emberizidae* | W,Sp,F | Neutral |
| DEJU | Dark-eyed Junco | *Junco hyemalis* | *Emberizidae* | All | Sensitive |
| WEME | Western Meadowlark | *Sturnella neglecta* | *Icteridae* | W | Sensitive |
| HOOR | Hooded Oriole | *Icterus cucullatus* | *Icteridae* | Sp,S,F | Tolerant |
| BUOR | Bullock's Oriole | *Icterus bullockii* | *Icteridae* | Sp,S | Sensitive |
| RWBL | Red-winged Blackbird | *Agelaius phoeniceus* | *Icteridae* | Sp,S,F | Neutral |
| BHCO | Brown-headed Cowbird | *Molothrus ater* | *Icteridae* | Sp,S,F | Neutral |
| BRBL | Brewer's Blackbird | *Euphagus cyanocephalus* | *Icteridae* | All | Sensitive |
| OCWA | Orange-crowned Warbler | *Leiothlypis celata* | *Parulidae* | W,Sp,F | Tolerant |
| NAWA | Nashville Warbler | *Leiothlypis ruficapilla* | *Parulidae* | F | Tolerant |
| MGWA | MacGillivray's Warbler | *Geothlypis tolmiei* | *Parulidae* | Sp | Sensitive |
| COYE | Common Yellowthroat | *Geothlypis trichas* | *Parulidae* | All | Neutral |
| YEWA | Yellow Warbler | *Setophaga petechia* | *Parulidae* | All | Sensitive |
| YRWA | Yellow-rumped Warbler | *Setophaga coronata* | *Parulidae* | W,Sp,F | Tolerant |
| BTYW | Black-throated Gray Warbler | *Setophaga nigrescens* | *Parulidae* | Sp | Sensitive |
| TOWA | Townsend's Warbler | *Setophaga townsendi* | *Parulidae* | W,Sp,F | Tolerant |
| WIWA | Wilson's Warbler | *Cardellina pusilla* | *Parulidae* | Sp,S,F | Sensitive |
| WETA | Western Tanager | *Piranga ludoviciana* | *Cardinalidae* | Sp | Sensitive |
| BHGR | Black-headed Grosbeak | *Pheucticus melanocephalus* | *Cardinalidae* | Sp,S | Sensitive |

## Appendix 1: Table 4. The probability (p^★^) that if an avian species was present at a site, it would be detected through the course of all surveys at a site during a season. Mean and 95% highest density interval (HDI).

|  |  | 95% HDI | |
| --- | --- | --- | --- |
| Season | Mean | lower | upper |
| Winter | 0.84 | 0.76 | 0.90 |
| Spring | 0.76 | 0.69 | 0.82 |
| Summer | 0.82 | 0.75 | 0.89 |
| Fall | 0.72 | 0.65 | 0.79 |

## Appendix 1: Table 5. Multi-species bird occupancy model results for each season, along with the proportion of species that had strong responses (defined as 75% HDI non-overlapping with zero). Significant logit coefficients (defined as 95% HDI non-overlapping with zero) are in bold. Italicized coefficients are those for which intervals include zero but there is a tendency toward one direction of effect.

|  | |  | 95% HDI | | Proportion of species responses | |
| --- | --- | --- | --- | --- | --- | --- |
| Model coefficient | | Mean | lower | upper | (-) | (+) |
| Spring model | |  |  |  |  |  |
|  | Intercept | -1.47 | -2.20 | -0.74 |  |  |
|  | **Distance from bay (km)** | **-0.52** | **-0.79** | **-0.27** | 0.52 | 0.01 |
|  | **Distance from stream (km)** | **-0.60** | **-0.91** | **-0.31** | 0.42 | 0.00 |
|  | Neighborhood canopy cover (%) | 0.05 | -0.29 | 0.39 | 0.15 | 0.18 |
|  | Neighborhood impervious cover (%) | 0.12 | -0.11 | 0.35 | 0.00 | 0.06 |
|  | **Local canopy cover (%)** | **0.29** | **0.05** | **0.53** | 0.01 | 0.25 |
|  | Local shrub cover (%) | 0.20 | -0.11 | 0.54 | 0.04 | 0.18 |
|  | Local native:non-native tree ratio | 0.07 | -0.20 | 0.35 | 0.04 | 0.15 |
|  | Local impervious cover (%) | 0.05 | -0.17 | 0.27 | 0.01 | 0.06 |
|  | Local vegetation structure (%) | -0.09 | -0.38 | 0.19 | 0.07 | 0.09 |
|  | Local native shrub cover (%) | 0.05 | -0.32 | 0.39 | 0.01 | 0.12 |
| Summer model | |  |  |  |  |  |
|  | Intercept | -0.95 | -1.91 | 0.03 |  |  |
|  | **Distance from bay (km)** | **-0.48** | **-0.81** | **-0.16** | 0.35 | 0.00 |
|  | **Distance from stream (km)** | **-0.41** | **-0.81** | **-0.04** | 0.33 | 0.04 |
|  | *Neighborhood canopy cover (%)* | *0.39* | *-0.03* | *0.83* | 0.11 | 0.28 |
|  | *Neighborhood impervious cover (%)* | *-0.28* | *-0.61* | *0.02* | 0.20 | 0.02 |
|  | Local canopy cover (%) | 0.15 | -0.13 | 0.46 | 0.04 | 0.17 |
|  | *Local shrub cover (%)* | *-0.33* | *-0.68* | *0.00* | 0.13 | 0.00 |
|  | Local native:non-native tree ratio | -0.09 | -0.39 | 0.21 | 0.11 | 0.02 |
|  | **Local impervious cover (%)** | **-0.28** | **-0.53** | **-0.04** | 0.33 | 0.00 |
|  | Local vegetation structure (%) | 0.15 | -0.21 | 0.53 | 0.07 | 0.24 |
|  | Local native shrub cover (%) | -0.13 | -0.62 | 0.30 | 0.04 | 0.09 |
| Fall model | |  |  |  |  |  |
|  | Intercept | -1.45 | -2.59 | -0.17 |  |  |
|  | Distance from bay (km) | -0.26 | -0.75 | 0.21 | 0.13 | 0.05 |
|  | **Distance from stream (km)** | **-0.51** | **-1.02** | **-0.05** | 0.18 | 0.03 |
|  | Neighborhood canopy cover (%) | -0.46 | -1.20 | 0.18 | 0.28 | 0.13 |
|  | Neighborhood impervious cover (%) | 0.01 | -0.39 | 0.37 | 0.00 | 0.00 |
|  | Local canopy cover (%) | -0.18 | -0.80 | 0.40 | 0.07 | 0.02 |
|  | **Local shrub cover (%)** | **0.71** | **0.16** | **1.42** | 0.00 | 0.37 |
|  | Local native:non-native tree ratio | -0.15 | -0.70 | 0.35 | 0.10 | 0.13 |
|  | Local impervious cover (%) | 0.31 | -0.17 | 0.87 | 0.02 | 0.20 |
|  | Local vegetation structure (%) | 0.51 | -0.10 | 1.34 | 0.02 | 0.22 |
|  | Local native shrub cover (%) | -0.11 | -1.53 | 1.29 | 0.02 | 0.00 |
| Winter model | |  |  |  |  |  |
|  | Intercept | -1.75 | -2.80 | -0.72 |  |  |
|  | **Distance from bay (km)** | **-0.48** | **-0.83** | **-0.15** | 0.38 | 0.05 |
|  | Distance from stream (km) | -0.09 | -0.37 | 0.18 | 0.12 | 0.09 |
|  | *Neighborhood canopy cover (%)* | *0.34* | *-0.04* | *0.72* | 0.07 | 0.29 |
|  | Neighborhood impervious cover (%) | -0.18 | -0.49 | 0.14 | 0.22 | 0.10 |
|  | **Local canopy cover (%)** | **0.28** | **0.03** | **0.55** | 0.00 | 0.19 |
|  | Local shrub cover (%) | 0.18 | -0.11 | 0.45 | 0.00 | 0.14 |
|  | Local native:non-native tree ratio | 0.06 | -0.27 | 0.36 | 0.05 | 0.14 |
|  | Local impervious cover (%) | -0.26 | -0.64 | 0.10 | 0.29 | 0.12 |
|  | Local vegetation structure (%) | -0.01 | -0.37 | 0.33 | 0.07 | 0.16 |
|  | Local native shrub cover (%) | -0.18 | -0.49 | 0.09 | 0.12 | 0.02 |

##

## Appendix 1: Table 6. Multi-species avian detection probability model results for seasonal occupancy models. Significant logit coefficients (defined as 95% HDI non-overlapping with zero) are in bold.

|  | |  | 95% HDI | |
| --- | --- | --- | --- | --- |
| Model coefficient | | Mean | lower | upper |
| Spring model | |  |  |  |
|  | Intercept | -1.37 | -1.84 | -0.94 |
|  | Julian date | -0.01 | -0.22 | 0.21 |
|  | Julian date quadratic | -0.02 | -0.13 | 0.09 |
|  | time after sunrise (min) | -0.02 | -0.12 | 0.07 |
|  | Noise (Leq) | -0.03 | -0.12 | 0.06 |
| Summer model | |  |  |  |
|  | Intercept | -0.94 | -1.48 | -0.49 |
|  | Julian date | -0.12 | -0.24 | 0.00 |
|  | Julian date quadratic | -0.05 | -0.16 | 0.07 |
|  | Time after sunrise (min) | 0.05 | -0.05 | 0.16 |
|  | Noise (Leq) | -0.08 | -0.17 | 0.02 |
| Fall model | |  |  |  |
|  | Intercept | -1.71 | -2.19 | -1.27 |
|  | Julian date | 0.19 | -0.06 | 0.45 |
|  | **Julian date quadratic** | **-0.15** | **-0.28** | **-0.02** |
|  | Time after sunrise (min) | -0.10 | -0.20 | 0.00 |
|  | Noise (Leq) | -0.01 | -0.12 | 0.10 |
| Winter model | |  |  |  |
|  | Intercept | -1.25 | -1.87 | -0.66 |
|  | Julian date | 0.18 | -0.14 | 0.51 |
|  | Julian date quadratic | 0.32 | -0.10 | 0.76 |
|  | Time after sunrise (min) | -0.04 | -0.13 | 0.05 |
|  | **Noise (Leq)** | **-0.12** | **-0.21** | **-0.02** |

| Appendix 1: Table 7. The predicted range of the number of bird species lost or gained (across values measured in our study) per each reported unit of change for said predictor. Only values derived from coefficients with HDIs that did not overlap zero are included. Full logit model summaries in Appendix 1: Table 5. | | | | |
| --- | --- | --- | --- | --- |
|  | Unit of change | Range of species lost or gained | | |
|  |  | Landscape | Neighborhood | Local |
| Spring Model | | | | |
| Distance from bay | 0.5 km | -0.8 to -1.8 | – | – |
| Distance from stream | 0.5 km | -2.7 to -8.3 | – | – |
| Local canopy cover | 10% | – | – | 1.1 to 1.56 |
| Summer Model | | | | |
| Distance from bay | 0.5 km | -0.7 to -0.9 | – | – |
| Distance from stream | 0.5 km | -1.8 to -3.9 | – | – |
| Local Impervious cover | 10% | – | – | -0.4 to -0.6 |
| Local shrub cover | 10% | – | – | -1.2 to -1.9 |
| Fall Model | | | | |
| Distance from stream | 0.5 km | -1.7 to -4.3 | – | – |
| Local shrub cover | 10% | – | – | 2.5 to 4.1 |
| Winter Model | | | | |
| Distance from bay | 0.5 km | -0.6 to -1.4 | – | – |
| Site canopy cover | 10% | – | – | 0.8 to 1.0 |

## Appendix 1: Figure 1. Avian community-level occupancy probability (a), detection probability (b), and species richness (c), by season. The 95% highest density interval of the posterior distribution of values are presented as error bars.


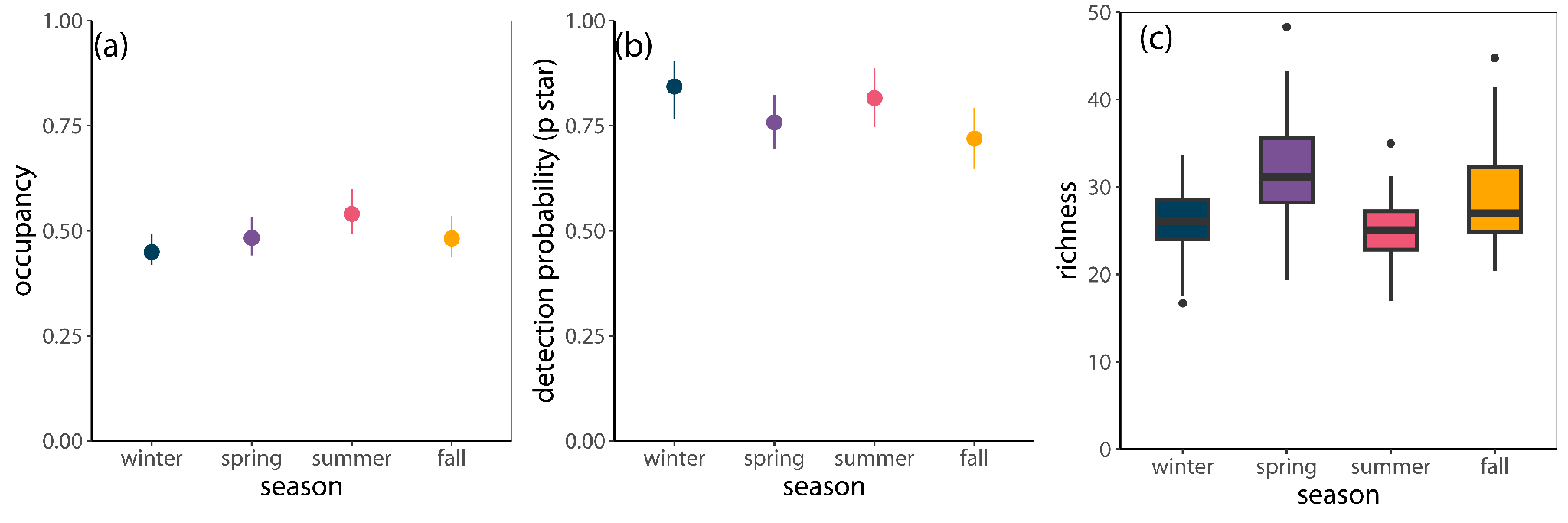


## Appendix 1: Figure 2. Bird occupancy probability by species and season. Species are sorted by their maximum mean occupancy probability. Error bars are the 95% highest density interval. See Appendix 1: Table 3 for species codes.

##
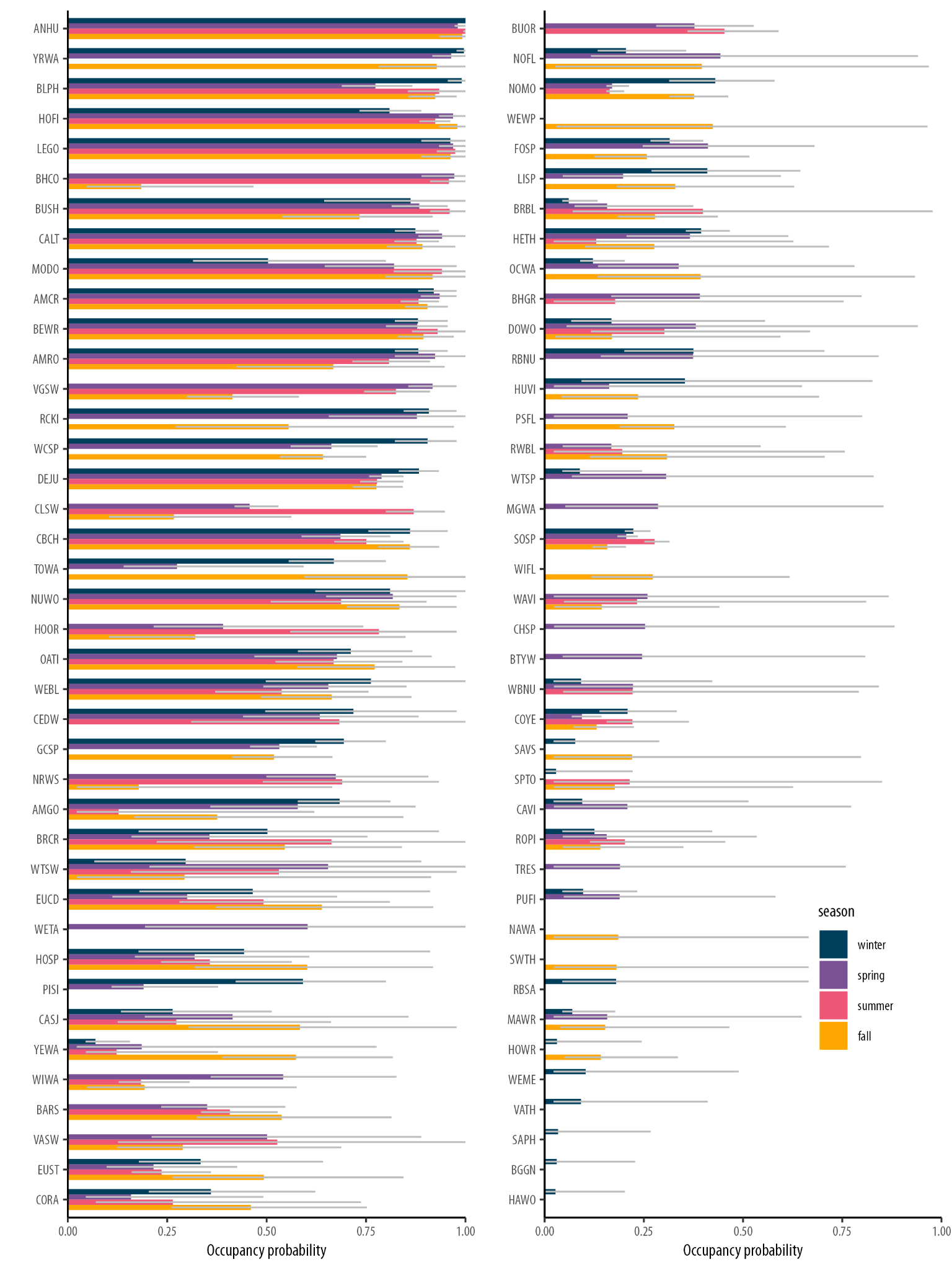


## Appendix 1: Figure 3. Avian detection probability by species and season. Species are sorted by their maximum mean detection probability. Error bars are the 95% highest density interval. See Appendix 1: Table 3 for species codes.
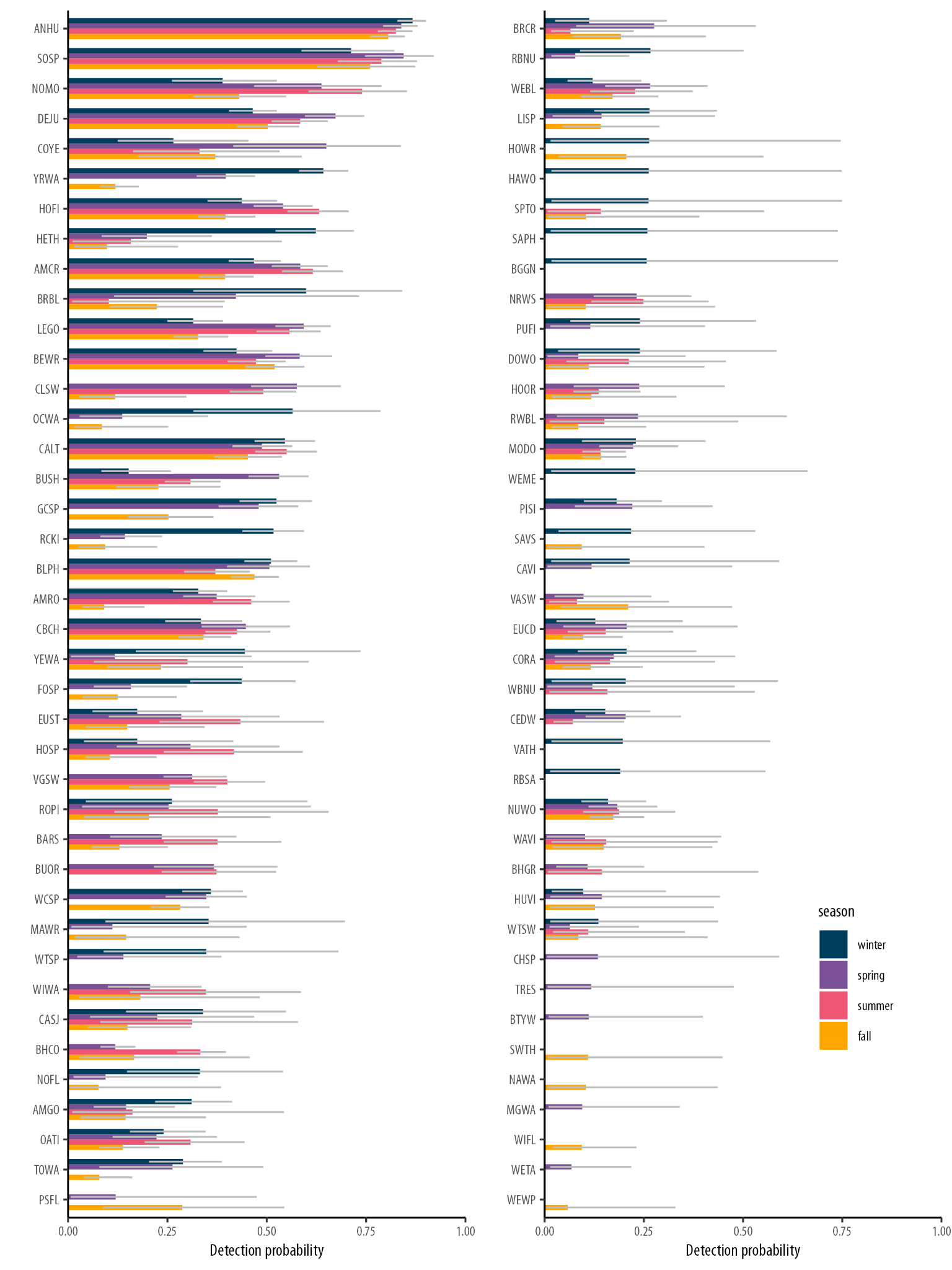


Appendix 1: Figure 4 Moran’s I results for bird occupancy models for each season: A) winter, B) spring, C) summer, D) fall. Each line represents the 97.5% HDI Moran’s I value for a species in
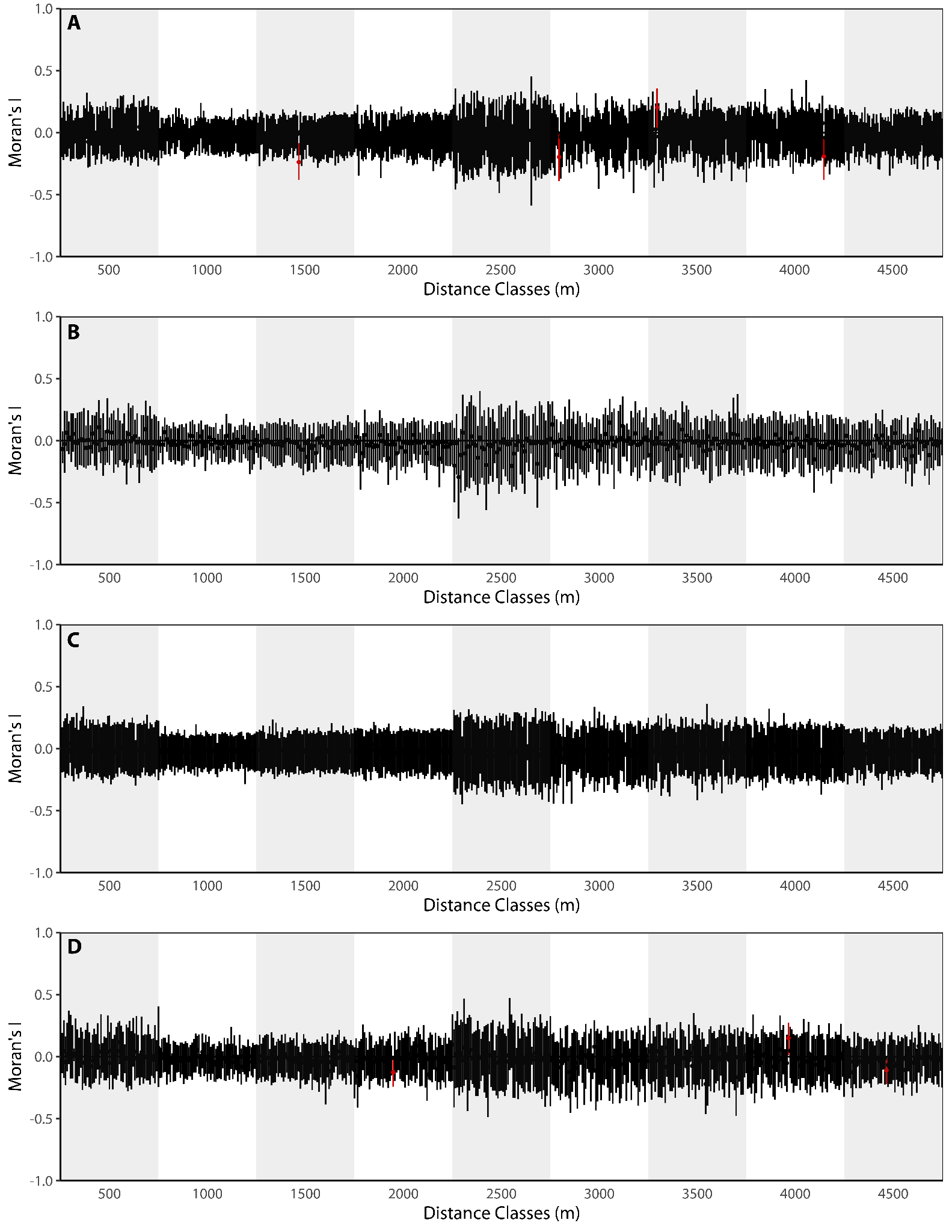
a distance class; red lines are values that significantly differ from zero (winter: n = 4; fall: n = 3).

Appendix 1:Literature Cited

Callaghan, C. T., W. K. Cornwell, A. G. B. Poore, Y. Benedetti, and F. Morelli. 2021. Urban tolerance of birds changes throughout the full annual cycle. Journal of Biogeography 48:1503–1517.

Pyle, P., and D. F. DeSante. 2003. Four-letter and six-letter alpha codes for birds recorded from the American Ornithologist’s Union check-list area. North American Bird Bander 28:64–79.
